# Nurses as guardians of time: the hidden clinical value of continuous care in geriatrics

**DOI:** 10.3389/fpubh.2026.1799845

**Published:** 2026-04-15

**Authors:** Yanyang Yang, Haihong Wang, Enci Li, Yaoyao Zheng, Yu Chen, Xiaoyan Wu

**Affiliations:** 1 Department of Geriatric Medicine, Wenzhou People’s Hospital, Wenzhou, China; 2 Department of Nursing, Wenzhou People’s Hospital, Wenzhou, China

**Keywords:** geriatric nursing, health trajectories, longitudinal monitoring, multimorbidity, predictive care

## Abstract

Ageing-related diseases are increasingly recognized as time-dependent processes characterized by gradual accumulation, fluctuation, and prolonged subclinical deterioration rather than discrete clinical events. However, prevailing geriatric care models remain largely episodic, limiting their capacity to detect early signals of decline and intervene proactively. This review synthesizes longitudinal evidence from epidemiology, ageing biology, and clinical nursing research to reconceptualize geriatric disease as a trajectory-based phenomenon. Geriatric nurses may play a particularly important role in monitoring longitudinal changes in older adults and may contribute to emerging predictive care approaches that aim to anticipate health deterioration and support earlier intervention. By integrating subtle physiological, functional, behavioral, and psychosocial changes over time, nursing practice enables trend-based clinical reasoning that extends beyond task execution toward predictive decision-making. Furthermore, we examine how emerging digital tools and time-series analytics can amplify, but not replace, nursing judgment in anticipatory care. Together, these perspectives position geriatric nursing as a frontline predictive system essential for transforming ageing care from reactive event management to proactive trajectory-informed intervention.

## 1 Introduction

The global demographic landscape is undergoing an unprecedented transformation driven by rapid population ageing (1, 2). The number of older adults continues to expand worldwide, and nearly half of those aged 60 years and older live with two or more chronic conditions, a phenomenon known as multimorbidity, with pooled prevalence estimates approaching 46% across diverse populations (3). Ageing is closely associated with a growing burden of non-communicable diseases including cardiovascular disease, cerebrovascular disorders, chronic respiratory conditions, diabetes, and neurodegenerative syndromes, which together account for substantial morbidity, mortality, and disability in older cohorts (4). In addition to the rising prevalence of chronic diseases, the sheer scale of health loss attributable to ageing is projected to continue increasing across regions, imposing sustained pressure on health systems and social care infrastructures (5). These converging demographic and epidemiological trends highlight that ageing populations are not only living longer, but are also living with increasingly complex health needs that span multiple physiological systems and disease processes.

Despite the clear evidence of rising multimorbidity and complexity in ageing populations, contemporary geriatric care models remain predominantly structured around episodic, event-driven approaches that emphasize isolated clinical encounters such as hospital admissions, exacerbations, or acute emergencies (6). These models, rooted in historical acute care paradigms, inadequately accommodate the longitudinal evolution of health and illness in older adults, particularly the slow transitions between states of function and dysfunction that occur outside formal clinical settings (7). Such episodic care fragmentation contributes to missed opportunities for early detection of deterioration, delayed interventions, and inefficient coordination among providers, ultimately undermining patient outcomes and satisfaction (8). Continuity of care research has long underscored that relational, informational, and management continuity is critical for older adults with complex needs, and that fragmented interactions are associated with gaps in care quality and poorer psychosocial outcomes (9, 10). Within this context, nursing practice too has been constrained by task-oriented workflows and discrete documentation processes that focus on isolated assessments rather than a comprehensive understanding of how an individual’s health unfolds over time. Consequently, nurses are often positioned as executors of prescribed tasks rather than as longitudinal interpreters of health trajectories, despite evidence that continuous engagement with patients enables richer understanding of risks and nuanced changes that predict future decline.

In contrast to snapshot-based care, a time-continuous perspective recognizes ageing and multimorbidity as inherently temporal processes characterized by gradual physiological deterioration, nonlinear fluctuations, and extended subclinical phases that precede overt clinical thresholds (11, 12). Longitudinal studies using advanced phenotyping, functional assessment, and trajectory analysis demonstrate that dynamic patterns of change in mobility, cognition, circadian physiology, and multimorbidity accumulation have stronger predictive validity for adverse outcomes than isolated measurements (13). These temporal signals, when systematically captured and interpreted, reveal critical inflection points where proactive interventions can alter disease courses, mitigate functional decline, and preserve independence (14, 15). Moreover, digital health technologies such as wearables and remote monitoring systems now enable high-resolution time series data that closely track real-world health trajectories, further amplifying the potential for continuous observation to inform predictive care strategies (16). Under this paradigm, nurses emerge as uniquely positioned to bridge the gap between longitudinal data and clinical action owing to their sustained, context-rich patient contact, capacity for trend interpretation, and central role in coordinating care across settings. This reframing elevates geriatric nursing from episodic task execution to trajectory-oriented surveillance and predictive risk orchestration, recognizing that the real value of care lies not in isolated interventions but in understanding and shaping health over time (17).

**Figure 1:**
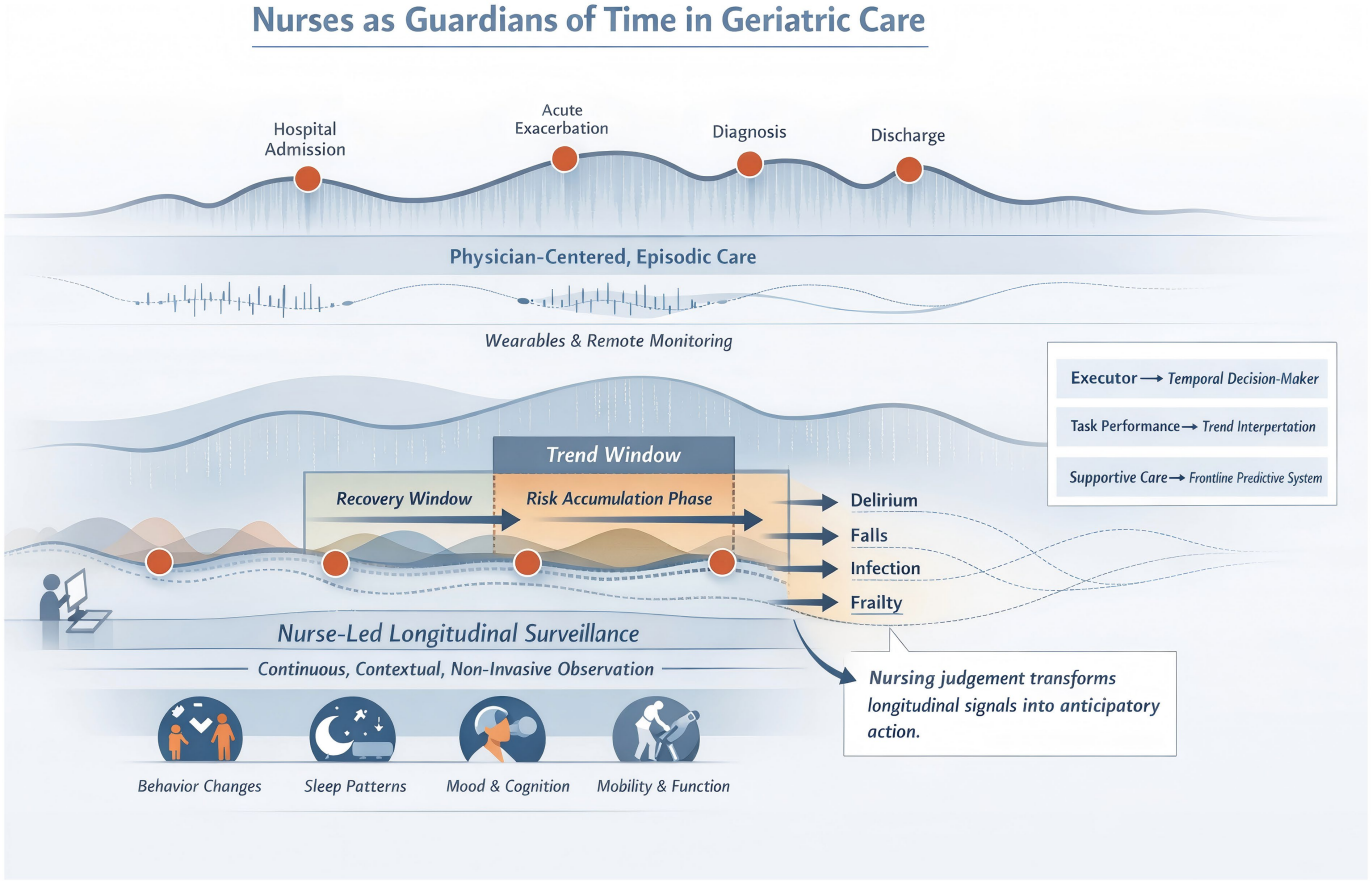
This schematic illustrates how nurse-led continuous surveillance transforms longitudinal patient signals into predictive clinical action, bridging gaps in episodic care and enabling early intervention in geriatric health trajectories.

This review synthesizes contemporary evidence at the intersection of ageing biology, longitudinal health measurement, nursing practice transformation, and digital health innovation to articulate a time-centric framework for geriatric nursing (Figure 1). We first examine how ageing and multimorbidity progress along continuous, heterogeneous trajectories and why episodic care models fail to capture the subtle but consequential early changes that presage clinical deterioration. Building on this conceptual foundation, subsequent sections reinterpret the role of nurses as longitudinal observers and integrators of temporal health information, delineating how continuous assessment tools, trajectory-based nursing metrics, and predictive analytics can extend nursing influence from monitoring to anticipatory intervention. We further explore the integration of smart technologies and interoperable data systems that enable real-time trend visualization and cross-professional coordination, supporting nurses’ capacity to translate complex temporal data into actionable care plans. Finally, the review considers future directions for geriatric nursing within a time-centric health system, highlighting implications for education, policy, ethical governance, and the development of nursing competencies that align with predictive, trajectory-informed care. Through this comprehensive synthesis, we aim to provide a conceptual and practical roadmap for aligning nursing practice with the temporal realities of ageing, thereby enhancing the predictive and preventive potential of care for older adults.

### 1.1 Literature identification and review approach

This article adopts an integrative narrative review approach aimed at synthesizing interdisciplinary evidence relevant to trajectory-oriented geriatric care, including ageing biology, multimorbidity research, geriatric nursing, and predictive health technologies. A structured literature search was conducted in PubMed, Web of Science, Scopus, and CINAHL covering publications from January 2000 to March 2025. Search terms included combinations of the following keywords: aging trajectory, multimorbidity progression, frailty dynamics, longitudinal ageing, geriatric nursing, predictive care, early warning systems, digital health in older adults. Studies were eligible if they: (1) investigated longitudinal ageing processes, frailty progression, or multimorbidity trajectories; (2) examined predictive monitoring or early detection of health deterioration in older adults; (3) addressed nursing roles in geriatric monitoring or trajectory-based care; (4) provided conceptual or empirical insights related to predictive or trajectory-oriented care models. Studies focusing exclusively on acute disease mechanisms without relevance to ageing trajectories or geriatric care were excluded. Titles and abstracts were screened for relevance, followed by full-text assessment of potentially eligible studies. Given the conceptual scope of this work, evidence was synthesized using a thematic integrative approach, emphasizing conceptual connections between biological ageing processes, clinical trajectory research, and nursing observation practices.

## 2 Geriatric diseases as time-dependent conditions

**Figure 2:**
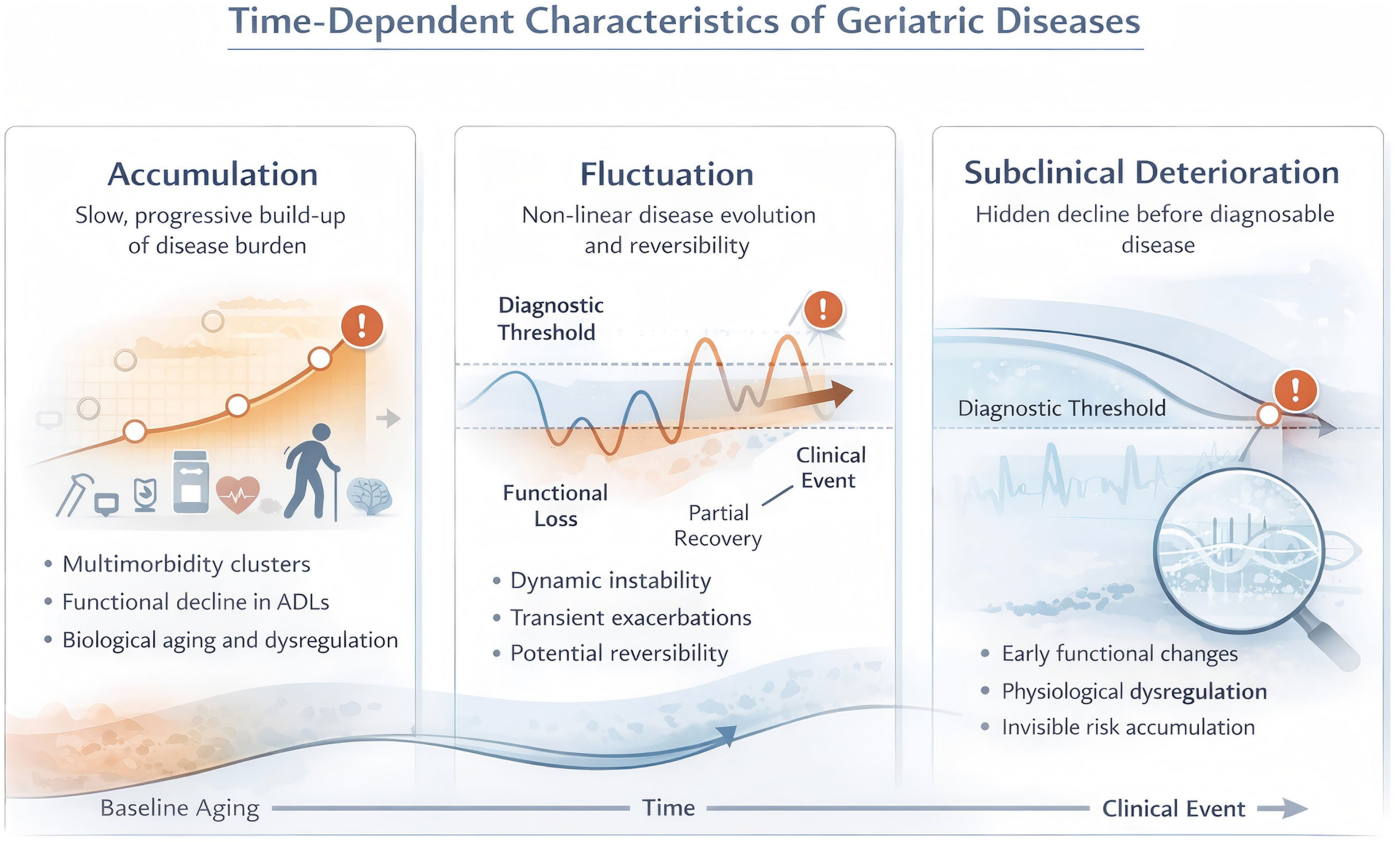
This figure illustrates geriatric diseases as time-dependent processes characterized by slow cumulative accumulation, nonlinear fluctuation with partial reversibility, and prolonged subclinical deterioration that precedes overt clinical events and escapes episodic care.

Geriatric diseases are increasingly recognized not as discrete clinical entities that emerge at identifiable moments, but as time-dependent processes that unfold across prolonged and heterogeneous trajectories. Unlike acute conditions, aging-related illnesses develop through the gradual accumulation of physiological dysregulation, interact dynamically across organ systems, and often fluctuate between states of vulnerability and relative stability long before diagnostic thresholds are reached. This temporal complexity fundamentally challenges care models grounded in episodic assessment and event-driven intervention, which are poorly equipped to capture slow, nonlinear, and subclinical changes. In this section, we conceptualize geriatric disease as a longitudinal phenomenon shaped by three interrelated temporal dimensions (Figure 2): the slow and cumulative build-up of disease burden over years, the presence of fluctuation and partial reversibility within disease trajectories, and the prolonged subclinical deterioration that remains invisible to snapshot-based care. Together, these features underscore why understanding aging and multimorbidity requires a trajectory-oriented perspective and why continuous, longitudinal observation is essential for anticipating decline and identifying meaningful windows for intervention.

### 2.1 The slow build-up of disease: why geriatric illnesses rarely begin at a single moment

The clinical progression of age-related chronic conditions is fundamentally a time-dependent process, characterized by a slow and cumulative accumulation of functional deficits and disease burden rather than abrupt onset. Unlike many acute illnesses (e.g., infections or trauma) with well-defined clinical start points, conditions common in older adults such as cardiovascular disease, metabolic disorders, neurocognitive decline, and musculoskeletal impairment tend to evolve over years to decades through subtle subclinical deterioration before reaching diagnostic thresholds (18). Epidemiological evidence robustly supports this view. In a 12-year longitudinal cohort study involving older adults aged ≥60, researchers identified distinct clinical trajectories of multimorbidity clusters that shifted dynamically over time, with individuals moving between disease patterns and experiencing corresponding changes in prognosis and mortality risk rather than experiencing sudden disease onset (19). This study demonstrated that multimorbidity is not static but accumulates and transforms across extended periods, reflecting the inherent time dependency of age-related conditions. Similar work using machine learning to classify multimorbidity progression in middle-aged and older individuals identified four distinct trajectory groups, ranging from stable low-risk disease burden to consistently deteriorating multimorbidity over years, confirming that the rate and pattern of chronic disease accumulation differ across aging populations and are shaped by underlying biological aging processes (20). In parallel, a nine-year study applying group-based trajectory modeling demonstrated that older adults could be stratified into distinct clusters based on the number of accumulating long-term conditions, with increasing age consistently associated with a higher burden of chronic disease, further underscoring that aging and chronic disease progression are inseparable temporal processes rather than sudden clinical events (21). From a clinical standpoint, this gradual accumulation is reflected in early functional changes that often precede formal diagnoses by years, creating a prolonged subclinical phase during which risk remains unrecognized in the absence of continuous monitoring (22). For instance, declines in gait speed, reductions in activities of daily living, loss of muscle mass, and alterations in sleep or appetite may occur long before thresholds for frailty or dementia are met (23). These changes, though subtle when viewed at a single time point, represent progressive physiological deterioration when tracked longitudinally and strongly correlate with later adverse outcomes. Moreover, multimorbidity does not simply represent the coexistence of discrete diseases; rather, it reflects interacting pathophysiological processes that progress together over time. Recent longitudinal imaging studies in older adults with elevated amyloid-*β* demonstrate that higher baseline multimorbidity burden is associated with faster accumulation of Alzheimer’s pathology over several years, linking peripheral chronic disease burden with neurodegenerative progression (24). Clinically, this suggests that the traditional snapshot paradigm, in which healthcare encounters detect disease only after symptom escalation, misses a critical window of preclinical progression during which interventions could potentially attenuate or delay further deterioration.

Crucially, the concept of gradual accumulation extends beyond disease counts to include temporal interdependence between lifestyle, risk factors, and disease progression. Longitudinal analyses of lifestyle patterns reveal that healthier behaviors in midlife associate with slower progression of chronic conditions into older age, whereas once multimorbidity is established, it can negatively influence subsequent lifestyle activities, creating feedback loops that accelerate decline (12). This interplay highlights an important clinical insight: early, sustained intervention on modifiable risk factors may alter an individual’s entire disease trajectory, emphasizing that time itself is a modifiable dimension of chronic illness progression. As clinicians and caregivers, recognizing that the path to multimorbidity and functional decline is often long and insidious, rather than sudden, shifts the focus of care from reactive episodic treatment to proactive longitudinal surveillance. In practical terms, this conceptual shift means that single-point clinical assessments, while necessary, are insufficient to capture the true evolution of chronic conditions in older adults (25). Instead, clinicians and care teams must adopt an integrative longitudinal perspective, where patterns and trajectories across multiple domains (physical function, cognitive status, disease accumulation) are monitored over time to reveal early signals of decline (26). Such longitudinal insights can identify individuals on adverse trajectories before irreversible damage occurs. For example, the identification of individuals who transition from a stable low-risk cluster to a progressively worsening multimorbidity trajectory should trigger earlier, tailored interventions aimed at preventing further decline (20). Increasingly, research highlights that integrating longitudinal disease trajectory information rooted in years of patient data into clinical decision-making improves risk stratification and may ultimately inform personalized prevention strategies that reflect each patient’s unique disease evolution rather than a one-size-fits-all approach (27, 28). In summary, chronic conditions in older adults do not typically emerge as isolated, acute events. Instead, they arise through a gradual, dynamic accumulation of physiological disruptions that unfold over extended time periods. This time-dependent nature of disease progression underscores the need for continuous, longitudinal clinical assessment to detect early changes and intervene before irreversible dysfunction ensues. Understanding multimorbidity and aging through this temporal lens not only aligns with emerging epidemiological evidence but also serves as a foundation for redefining clinical care pathways that prioritize proactive, trajectory-informed management for older adults.

### 2.2 Fluctuation and reversibility in disease trajectories

The clinical progression of many geriatric syndromes is nonlinear and dynamic, marked by fluctuation and, under certain conditions, partial reversibility of pathological states rather than inexorable decline. Longitudinal evidence from high-impact research demonstrates that states commonly associated with aging, including frailty, multimorbidity clusters, and subclinical dysregulation, can shift over time, underscoring the importance of appreciating chronic disease progression as a trajectory with multiple possible paths rather than a fixed end point (29, 30). For example, a 15-year longitudinal study in older adults from the Swedish SNAC-K cohort demonstrated that adherence to health-promoting dietary patterns was associated with slower accumulation of chronic diseases over time, whereas pro-inflammatory diets accelerated multimorbidity progression, illustrating that disease trajectories are dynamic and modifiable rather than predetermined (31). Molecular-level evidence further illustrates the temporal complexity of geriatric diseases. Large-scale plasma proteomic analyses have revealed that molecular signatures associated with neurodegenerative diseases such as Parkinson’s disease change years before clinical diagnosis, with specific proteins showing continuous decline long before motor symptoms emerge (32). These patterns indicate that the biological processes underlying clinical symptoms unfold over extended periods and that preclinical pathophysiological changes may be detectable long before overt clinical manifestation. In parallel, imaging and physiological phenotyping from the UK Biobank has shown that biological aging does not occur at a constant rate across organ systems; instead, aging trajectories differ by organ type and interact dynamically, such that age acceleration in one system can precipitate systemic effects and alter subsequent disease progression (33). This heterogeneous aging across systems supports the clinical observation that older adult patients’ functional status may deteriorate rapidly after a seemingly quiescent period or partially improve with targeted interventions, reflecting periods of relative reversibility embedded within a broader trajectory of decline.

The concept of fluctuation in geriatric disease is supported by longitudinal studies of physiological rhythms and frailty. In a cohort of over 1,000 older adults followed annually for up to 16 years, disrupted circadian rest-activity patterns were linked to not only the onset but also a faster progression of frailty over time, with rhythm instability and variation correlating with decline in muscle strength and body composition changes that are central to frailty progression (34). Importantly, these associations were evident well before frailty was clinically diagnosed, indicating that temporal fluctuations in circadian physiology represent early indicators of future functional trajectory shifts. Such findings underscore that geriatric syndromes like frailty do not proceed in a simple, linear fashion, but rather reflect ongoing interplay among multiple physiological domains whose fluctuation patterns over time can herald transition points in clinical status. The clinical implications of these fluctuating trajectories extend beyond single diseases to multimorbidity, functional capacity, and health behavior interactions over time. Recent longitudinal analyses of multimorbidity patterns in large populations have identified transitions from less complex disease profiles to multisystemic chronicity, with socioeconomic factors and other determinants influencing the direction and speed of these changes (6). Similarly, studies of lifestyle factors such as integrated physical activity and health behaviors have shown associations with slower cognitive decline across genetically susceptible groups, indicating that the slope of functional trajectories can be modulated by modifiable factors even in later life (35). These emergent data emphasize that clinical trajectories in older adults are shaped by both intrinsic biological processes and external influences, creating windows of opportunity for intervention that can alter the course of decline, postpone adverse events, or even achieve meaningful improvements in health status.

Taken together, the evidence from diverse publications converges on a core insight: geriatric disease progression is inherently dynamic, exhibiting periods of fluctuation and potential partial reversibility that are detectable over time when measured longitudinally. Rather than viewing aging-related conditions as steadily downward in trajectory, clinicians and caregivers should recognize the temporal nature of disease courses, where variability in disease markers, functional metrics, and organ-specific aging patterns may signal clinically meaningful opportunities for intervention. Understanding these complex time-dependent trajectories is essential for designing predictive care models, prioritizing preventive strategies, and optimizing individualized management plans. By integrating serial assessments and recognizing temporal inflection points, health professionals can better anticipate clinical deterioration, tailor care sequences more effectively, and contribute to improved outcomes and quality of life for older adults navigating multimorbidity and age-related functional decline.

### 2.3 Subclinical deterioration and the blind spots of episodic care

Subclinical deterioration represents the early, often imperceptible physiological and functional changes that precede diagnosable clinical syndromes in older adults, and understanding these subtle changes is critical to reframing geriatric care around time-based trajectories rather than discrete episodes. For example, an 11-year cohort study among Chinese older adults revealed distinct joint trajectories of physical frailty and cognitive change, many of which began before overt disability or clinical impairment was evident, demonstrating that subtle shifts in cognition and frailty risk stratify mortality risks over time (36). Physiological dysregulation, a multisystem biological construct measured via longitudinal biomarker arrays, has also been shown to predict mortality, cognitive decline, and disability beyond traditional frailty scales, further establishing that biological signals of decline precede clinical events by years (37). At the cellular level, the accumulation of senescent cells and the associated senescence-associated secretory phenotype (SASP) have been identified as fundamental drivers of systemic deterioration that are not captured in conventional clinical encounters until manifesting as disease (38). From a functional perspective, research in community-dwelling older adults has shown that early changes in mobility and balance predict subsequent cognitive impairment and dementia, even when performance remains within normal clinical limits at initial assessment (39). Animal models further support this concept of subclinical deterioration: cross-sectional lifespan studies in mice demonstrate that declines in gait and metabolic function begin well before overt frailty or disease, indicating early physiological compromises that mirror human subclinical changes (40). Within the realm of brain aging, population and animal neuroscience research highlights that molecular and network alterations in neural circuits manifest prior to cognitive diagnoses, reinforcing that subclinical deterioration is a multiscale process spanning molecules to behavior (41). Finally, evidence from longitudinal frailty transition studies reveals that changes in frailty status often occur independently of overt clinical disease progression, emphasizing that subclinical shifts in resilience and functional capacity may be more informative than episodic clinical events alone (42). Collectively, these examples illuminate how subclinical deterioration occurs across systems and scales, from cellular senescence and physiological dysregulation to mobility patterns and cognitive trajectories, and how such changes escape detection in traditional care models focused on episodic events.

Recognizing subclinical deterioration as a measurable and clinically meaningful stage of aging challenges the prevailing assumption that care should begin only after disease thresholds are crossed; instead, it suggests that care strategies must pivot toward trend recognition and prediction based on longitudinal data across biological, functional, and clinical dimensions. Blood-based molecular biomarkers, including miRNAs linked with inflammation and energy homeostasis, have been associated with frailty phenotypes even in the absence of overt clinical frailty, indicating avenues for early stratification of at-risk individuals that episodic care overlooks (43). Consensus conceptual frameworks such as “cognitive frailty,” which integrate physical and cognitive subclinical impairments, have been proposed precisely to capture the early multisystem decline that prefigures dementia and disability, reinforcing the need for multidomain monitoring rather than isolated snapshot assessments (44). Aging biology frameworks also emphasize that mitochondrial dysfunction, DNA damage responses, and loss of proteostasis occur at the molecular level across decades, preceding clinical manifestations and requiring longitudinal biomarker tracking to be detected early (45). Experimental geroscience platforms are advancing measures of aging *in vivo* that isolate subtle biological shifts in processes such as autophagy and oxidative stress, revealing pathways where early decline is measurable yet subclinical (46). These subclinical signatures are not benign; rather, they correlate with higher risk for frailty progression, loss of resilience, and increased vulnerability to stressors—domains where nurse-led continuous surveillance is uniquely positioned to observe and act across time. In this context, the frequent absence of early warning signs in episodic care represents a systemic blind spot: nurses, through longitudinal functional assessment and trend interpretation, can bridge the gap between molecular insight and clinical practice by continuously contextualizing subtle multi-system changes that would otherwise remain latent until irreversibly progressed.

Importantly, evidence from human cohort studies provides direct support for the concept that health deterioration in older adults unfolds as gradual and measurable trajectories rather than abrupt clinical events. Large longitudinal analyses of community-dwelling older adults have demonstrated that functional indicators such as gait speed, mobility, and physical performance strongly predict long-term outcomes including disability, hospitalization, and mortality. For example, a pooled analysis of nine cohort studies including more than 34,000 adults aged ≥65 years showed that gait speed was strongly associated with survival, with faster gait speeds predicting substantially longer life expectancy (47). These findings suggest that subtle functional changes can reflect multisystem physiological decline years before major clinical events occur. Similarly, longitudinal geriatric cohort studies have shown that trajectories of frailty, multimorbidity, and functional decline evolve progressively across the life course and can be detected through repeated clinical assessments (48). Importantly, many of these assessments, including physical function measurements, symptom monitoring, and functional status evaluation, are commonly performed within routine nursing or multidisciplinary geriatric care. Together, these human observational studies provide real-world clinical evidence that ageing-related deterioration follows identifiable trajectories in older populations, supporting the relevance of continuous monitoring and early trajectory recognition in geriatric care.

## 3 Nursing as longitudinal surveillance: the unique temporal role of nurses in geriatric care

The progression of age-related conditions is inherently temporal, characterized by gradual deterioration, periodic fluctuations, and subclinical shifts that manifest long before traditional clinical thresholds are reached (49). This temporal complexity creates a landscape in which nurses play a uniquely indispensable role in longitudinal surveillance, leveraging frequent patient contact, context-embedded observation, and non-invasive assessments to detect early warning signals indicative of adverse trajectories (50). Longitudinal cohort research in aging populations such as the English Longitudinal Study of Ageing (ELSA) illustrates the profound value of serial, nurse-delivered evaluations; in ELSA, trained nurses perform repeated physical function measurements, anthropometry and biomarker collection across multiple waves spanning decades, enabling nuanced tracking of functional and health trajectories that correlate with later adverse outcomes (51). Similarly, nurse-led fall prevention programs consistently demonstrate that structured longitudinal monitoring and intervention reduce the incidence of falls, a leading cause of morbidity among older adults, by continuously detecting evolving risk factors such as gait instability, balance changes, and environmental triggers over time (52). These longitudinal surveillance processes allow nurses to integrate multi-dimensional data across time, identifying early deviations from baseline in behavior, mobility, sleep, and appetite that traditional episodic assessments frequently miss. Several empirical studies underscore the operationalization of this nurse-led temporal advantage in diverse clinical settings. Frail older adults participating in proactive primary care programs report that nurses’ monitoring role in assessing physical, cognitive, and social risk factors over repeated visits is central to anticipating change and tailoring care plans that preempt crises (53). In acute care, the CHRONOFALLS multicentre program implemented by nurse teams used temporal analyses of fall patterns (chronopreventive measures) to substantially reduce in-hospital fall rates by recognizing time-linked risk patterns and adapting preventive strategies accordingly (54). Quasi-experimental research in Ethiopia evaluating a nurse-led intervention for rural older adults further illustrates that serial assessments of frailty, nutrition, activities of daily living, and mood over multiple time points enable detection of health decline and the tailoring of care interventions that are contextually relevant to community realities (55).

These examples collectively demonstrate that nurses are uniquely positioned to transform temporal health data into actionable clinical insights. Nurses’ high-frequency interactions allow them to observe micro-level fluctuations in behavior and function, such as progressive slowing in mobility or subtle appetite changes that, when tracked longitudinally, predict downstream events like hospitalization or loss of independence. For instance, studies on nurse-led fall prevention have shown that ongoing assessment of balance, strength, and environmental risk factors, combined with education and behavior modification, can significantly reduce injurious fall rates and change patient behavior over time, thereby linking surveillance to improved clinical outcomes (56). Likewise, nurse-managed comprehensive geriatric assessments extend across care settings, with serial evaluations incorporated into inpatient, outpatient, and home care to monitor functional resilience and response to interventions, supporting decision-making that is informed by temporal trends rather than isolated snapshots (57). In qualitative studies of specialist geriatric nurses, practitioners report that interpreting serial changes in cognition, mood, and engagement across shifts is critical for early detection of delirium and other acute deteriorations, exemplifying how nurse-embedded observation captures fluctuation and context that episodic clinical assessments cannot (58). The temporal integration that nurses provide is not limited to physical function: repeated longitudinal contact enables detection of changes in sleep patterns, nutritional intake, mood disturbances, and social engagement, all of which are potent early indicators of adverse health trajectories (59). In community trials, nurse-led multifaceted group programs that track functional capacity, metabolic indicators, and social involvement over multiple assessments demonstrate that surveillance over time can both identify early deterioration and guide individualized preventive strategies (60). Even outside traditional nursing frameworks, research on nurse-led perioperative services for older surgical patients emphasizes that repeated frailty assessments before, during, and after surgery improve risk stratification and postoperative recovery planning, further highlighting the temporal value nurses bring across clinical phases (61). Thus, nursing longitudinal surveillance encompasses both early detection of subtle changes and the dynamic adaptation of care plans, ensuring that interventions are temporally aligned with each patient’s unique aging trajectory.

In summary, the role of nurses in geriatric longitudinal surveillance is anchored in repeated, context-rich observations that bridge the gap between subclinical deterioration and overt disease progression. Through high frequency contact, embedded assessment, and interpretation of temporal patterns across multiple domains including behavioral, functional, physiological, and psychosocial dimensions, nurses transform longitudinal surveillance from passive record keeping into predictive clinical action. This nurse-led temporal perspective not only enhances early detection and prevention but also reframes nursing as a core decision-making function within geriatric care systems, directly addressing the limitations of episodic care and supporting outcomes that reflect sustained well-being and resilience.

## 4 From monitoring to prediction: how continuous nursing observation enables predictive care

**Figure 3:**
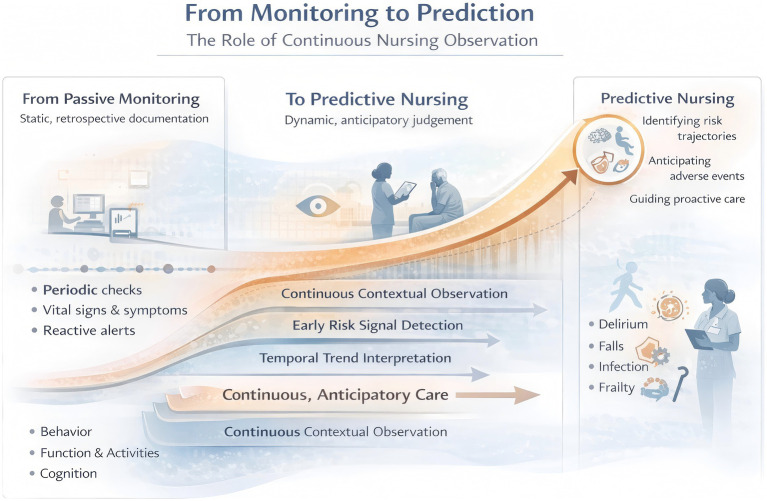
From monitoring to prediction in geriatric nursing: continuous, nurse-led longitudinal observation transforms fragmented clinical data into time-aware trend recognition, enabling early risk anticipation and proactive intervention rather than retrospective response.

As geriatric care increasingly confronts complexity, uncertainty, and prolonged disease trajectories, the clinical value of nursing observation can no longer be confined to passive monitoring or retrospective documentation. Instead, continuous nursing observation must be understood as a forward-looking, time-aware process that enables anticipation rather than reaction. This chapter advances the argument that predictive care in geriatrics emerges not from isolated data points or automated alerts alone, but from the integration of longitudinal observation, early risk signals, and clinical reasoning grounded in temporal context (Figure 3). We first reconceptualize continuous nursing observation as an interpretive surveillance process with inherent predictive capacity. We then examine how nurses’ sensitivity to subtle preclinical changes allows early identification of evolving risk trajectories across physiological, functional, and behavioral domains. Finally, we synthesize these insights into a time-series–based clinical reasoning model of predictive nursing, in which nursing judgment serves as the critical mechanism that transforms longitudinal data into anticipatory action. Together, these perspectives position nurses not merely as monitors of patient status, but as active agents in forecasting deterioration and shaping proactive, trajectory-informed care for older adults.

### 4.1 Beyond passive monitoring: reframing continuous nursing observation

In traditional clinical practice, nursing observation and patient monitoring have often been equated with periodic measurement and documentation of vital signs and clinical parameters at discrete time points (62). This view inherently constrains nursing to a reactive or snapshot-based role, whereby care decisions are triggered only after pathological thresholds are crossed (63). However, emerging evidence from cutting-edge clinical research challenges this paradigm. In the context of ageing populations and complex multimorbidity, continuous nursing observation must be redefined not as mere repetition of discrete observations but as a dynamic, context-embedded surveillance process with interpretive and predictive value (64). This reframing has profound implications for improving outcomes in older adults, where early subclinical changes often precede overt deterioration by hours or days and where nurses are uniquely positioned to integrate temporal trends with clinical insight (65). Recent translational research published in Nature Medicine exemplifies how nursing-driven continuous surveillance can be harnessed to predict clinical deterioration in real time. The COmmunicating Narrative Concerns Entered by RNs (CONCERN) Early Warning System (EWS) was developed to capture patterns in nurses’ documentation that reflect evolving patient risk and to transform these patterns into actionable risk scores (66). In a pragmatic, cluster-randomized controlled trial involving nearly 61,000 hospital encounters, the CONCERN EWS significantly reduced instantaneous mortality risk by 35.6% and length of stay by 11.2% when integrated into clinical care workflows, compared with usual practice. Secondary outcomes, including sepsis risk, also improved, highlighting the potential of modeling nursing surveillance as a real-time predictive tool rather than solely an administrative record of events. To fully appreciate the distinction between passive monitoring and interpretive continuous observation, it is instructive to consider the limitations of traditional monitoring systems. Conventional Early Warning Scores (EWS) such as MEWS and NEWS are calculated at discrete intervals and largely depend on instantaneous vital sign thresholds to trigger alerts; they often fail to account for trajectory information, which is the pattern of change over time. Continuous autonomous systems show promise in detecting physiological changes, but their utility remains constrained when they lack clinical context and interpretive frameworks (67). Real-time predictive systems such as CONCERN provide a model for integrating time-series patterns of clinical observations with nursing judgment, thereby enhancing situational awareness and enabling timely escalation of care.

A core attribute of this reframed conceptualization is the shift from static risk thresholds to trend-based interpretation. Nurses inherently perform this trend analysis through ongoing assessments that compare current findings with historical baselines (68). For example, a gradually increasing respiratory effort or subtle alterations in cognition over successive assessments may not breach standard physiological cutoffs but could signal impending decompensation (69). When formalized through predictive algorithms, such trends can be quantified and contextualized, allowing for early interventions that preempt crisis events. The value of trend analysis has been echoed in broader predictive analytics research: in non-nursing contexts, multimodal machine learning models that integrate continuous physiological data have demonstrated improved prediction of clinical decompensation events compared with models relying on static cross-sectional features (70). Equally important is the recognition that continuous nursing observation operates within a contextual framework that is qualitatively different from automated sensor data. Nurses synthesize information from multiple domains, including physiological, behavioral, and psychosocial factors, and understand how these domains interact in the context of an individual patient’s baseline and trajectory (71). This synthesis cannot be replicated by raw data streams alone. Even advanced continuous monitoring devices require human interpretation to contextualize trends against individual and situational baselines. In the predictive health informatics domain, models that solely rely on physiological waveform or vital sign data demonstrate the technical feasibility of early risk detection, but they still often require clinician interpretation in clinical practice due to variability across patients and clinical conditions (72).

In summary, the emerging evidence from predictive surveillance research, especially exemplified by interventions like the CONCERN EWS, mandates that continuous nursing observation be redefined beyond passive monitoring to an interpretive, *time-aware clinical reasoning process*. This reframing highlights the unique value nurses bring to geriatric care: their continuous presence and longitudinal judgments are not administrative artifacts but essential predictive signals that, when leveraged systematically, can anticipate deterioration and guide timely interventions. As health systems evolve in response to ageing populations and complex chronic disease patterns, integrating interpretative continuous nursing surveillance into predictive clinical models will be central to realizing safer, more proactive care.

### 4.2 Early signals and risk trajectories: nursing sensitivity to preclinical deterioration

Emerging evidence from longitudinal aging research indicates that early physiological, behavioral, and functional deviations often precede clinically overt deterioration by months to years, making them critical targets for prediction and preventive care. For example, disturbances in circadian rest activity rhythms, measurable through wearable actigraphy, have been prospectively associated with incident frailty and accelerated frailty progression in older adults (34). These findings suggest that altered sleep wake patterns may serve as early indicators of declining physiological resilience well before frailty becomes clinically manifest. Similarly, gut microbial and circulating metabolomic signatures correlate with frailty severity, and composite microbial scores significantly predict near-term mortality, underscoring the role of microbiome–host interactions as early risk signals of multisystem decline (30). Longitudinal cohort analyses further demonstrate that transitions between functional health states are heterogeneous, with distinct trajectories of symptom clusters (e.g., pain, mobility limitation, fatigue) showing strong associations with future disability and functional decline, signalling the dynamic nature of preclinical deterioration (73). These findings support the concept that trajectory-based indicators, which reflect gradual changes in function or physiology, are more predictive than single point measurements.

Beyond frailty trajectories, several preclinical markers have been linked to common geriatric adverse outcomes, offering nurses concrete signals to inform predictive care. First, prolonged sleep duration variation or fragmentation correlates bidirectionally with frailty progression, implying that even mild alterations in sleep patterns can herald functional decline (74). Second, multidimensional motor parameters such as gait variability and reduced stride length predict increased frailty risk, reflecting early neuromuscular compromise (75). Third, multimorbidity trajectories identified by clustering approaches reveal patterns of health deterioration that can stratify future risk profiles, enabling early identification of individuals on high-risk paths (20). Fourth, disruptions in glucose metabolism, as reflected by decreased estimated glucose disposal rates, have been associated with imminent frailty progression and may signal metabolic dysregulation preceding overt disability (76). Fifth, delirium risk is strongly linked to pre-existing frailty and sarcopenia, with changes in attention, motor performance, and nutritional status serving as early behavioral markers of neurocognitive vulnerability (77). The acute and fluctuating course of delirium makes its early detection vital, as it predicts prolonged hospitalization and adverse outcomes; subclinical attentional fluctuations may precede full-blown syndromes (78). Sixth, visual and sensory impairments in older populations track with increased risks of falls and functional loss, often manifesting before formal clinical recognition (79). Seventh, polygenic risk markers, including variants linked to lipid metabolism and immunoinflammatory pathways, have been shown to correlate with frailty phenotypes, suggesting that genetically determined biological risk signatures may also serve as early predictors (80). Eighth, upward deviations in routine blood biomarkers such as white blood cell count or metabolite ratios have been associated with higher frailty indices, indicating subclinical inflammation and metabolic dysregulation as early harbingers of health decline (30). Ninth, rates of change in grip strength and body mass index correlate with accelerated decreases in physical resilience, revealing that subtle longitudinal declines in muscle function or nutritional status often precede overt disability (34). Finally, community-based longitudinal studies underscore that change trajectories in functional disability (e.g., rising difficulty in instrumental activities of daily living) can forecast future dependence and institutionalization, illustrating the practical clinical value of monitoring functional signals over time (73). Collectively, these signals spanning circadian patterns, microbiome shifts, metabolic markers, neuromuscular dynamics, cognitive fluctuations, sensory decline, genetic predispositions, inflammatory markers, muscle function changes, and disability trajectories exemplify how early, often non-threshold changes can be integrated into a risk trajectory framework that surpasses conventional episodic assessments.

### 4.3 Predictive nursing in geriatrics: a time-series-based clinical reasoning model

Building on the reframing of nursing observation as a dynamic, context-embedded process and the recognition of early risk trajectories, predictive nursing posits that temporal trends across multiple health domains, rather than isolated point measurements, offer the most clinically actionable insights for anticipating deterioration in older adults (31). This perspective aligns with the broader shift in clinical informatics toward longitudinal modeling using electronic health records and time-series data to forecast outcomes such as mortality, disability, and care needs over extended horizons (81). For instance, attention-based deep learning models leveraging longitudinal primary care data from over 1.4 million older individuals demonstrated that incorporating the temporal sequence of clinical events substantially improves prediction accuracy for one-year and five-year all-cause mortality, nursing home admission, and home care requirements, outperforming models that only consider static historical snapshots (82). These findings emphasize that nursing models which systematically integrate temporal patterns into clinical reasoning can more reliably identify risk states that would otherwise remain obscured within isolated assessments. In this light, predictive nursing does not merely quantify risk as a binary threshold event; it interprets the direction, acceleration, and interactions of evolving physiological, functional, and behavioral indicators to inform anticipatory care. It is important to note that the conceptual framework proposed in this review integrates insights from multiple levels of evidence, including ageing biology, multimorbidity trajectory research, clinical monitoring systems, and emerging predictive analytics. While these domains collectively highlight the importance of temporal patterns in health decline, they represent heterogeneous evidence sources. Therefore, the present framework should be interpreted as a conceptual synthesis intended to stimulate further empirical investigation rather than as a fully validated clinical model.

Crucially, predictive nursing builds upon but extends beyond statistical or machine learning risk models by incorporating clinical reasoning and nursing judgment as key interpretive elements. Predictive models developed within geriatric and home healthcare contexts further illustrate how time series approaches can be embedded into clinical workflows. The inclusion of nursing-specific variables, such as length and character of nurse visits, underscores that nursing care itself contributes meaningful temporal signal beyond traditional biomedical indicators. Likewise, predictive models for postoperative frailty in older adults have been successfully constructed using diverse predictors that span demographic, perioperative, and clinical characteristics, offering validated tools for early identification of individuals at elevated risk following surgical interventions (83). These examples highlight that predictive nursing can synergize time-series data analytics with nurses’ longitudinal observations—transforming raw temporal data into clinical judgments that foresee decline and enable tailored interventions. From a practical and theoretical standpoint, then, predictive nursing represents a clinical reasoning model that synthesizes trend analysis, temporal contextualization, and decision activation into a coherent approach for managing complexity in geriatric care (84). Unlike classical predictive models focused solely on algorithmic risk scores, predictive nursing emphasizes the interpretive integration of diverse data streams, encompassing physiological measurements, functional performance, behavioral patterns, and care process variables, into a holistic temporal narrative of risk. For example, when multimodal models incorporate behavioral and functional trend indicators such as cadence changes, activity shifts, or early agitation signals detected via sensors and interpreted within clinical context, they can outperform static risk tools for specific outcomes like early behavioral deterioration in dementia populations. Similarly, pilot predictive frameworks utilizing machine learning to identify synergistic effects of multiple geriatric syndromes on quality-of-life outcomes demonstrate that considering interacting longitudinal patterns across domains yields more comprehensive risk stratification (85). By situating nursing judgment at the nexus of these predictive processes, predictive nursing empowers clinicians not only to forecast adverse events, but to interpret when and how trends should trigger tailored preventive strategies. In practice, this means continuously adjusting the care trajectory based on a nurse’s synthesis of trend information—thereby pre-empting crises and optimizing patient outcomes.

It is important to clarify the scope and nature of the framework proposed in this review. The evidence discussed throughout this article, including findings from ageing biology, multimorbidity trajectory research, predictive monitoring systems, and digital health technologies, serves primarily to illustrate the growing recognition that health decline in older adults unfolds along longitudinal and heterogeneous trajectories. Building upon this interdisciplinary evidence base, we propose a conceptual interpretation in which nursing practice functions as a form of trajectory-oriented clinical reasoning grounded in continuous observation and temporal pattern recognition. This conceptualization should therefore be understood as an evidence-informed synthesis rather than a validated predictive model. The purpose of integrating these diverse evidence domains is not to review each field exhaustively, but to highlight converging insights that support the central argument of this review: that nurses, through sustained patient contact and longitudinal observation, occupy a uniquely positioned role in recognizing early trajectory changes and enabling anticipatory geriatric care.

## 5 Clinical implications: redesigning geriatric nursing practice

**Figure 4:**
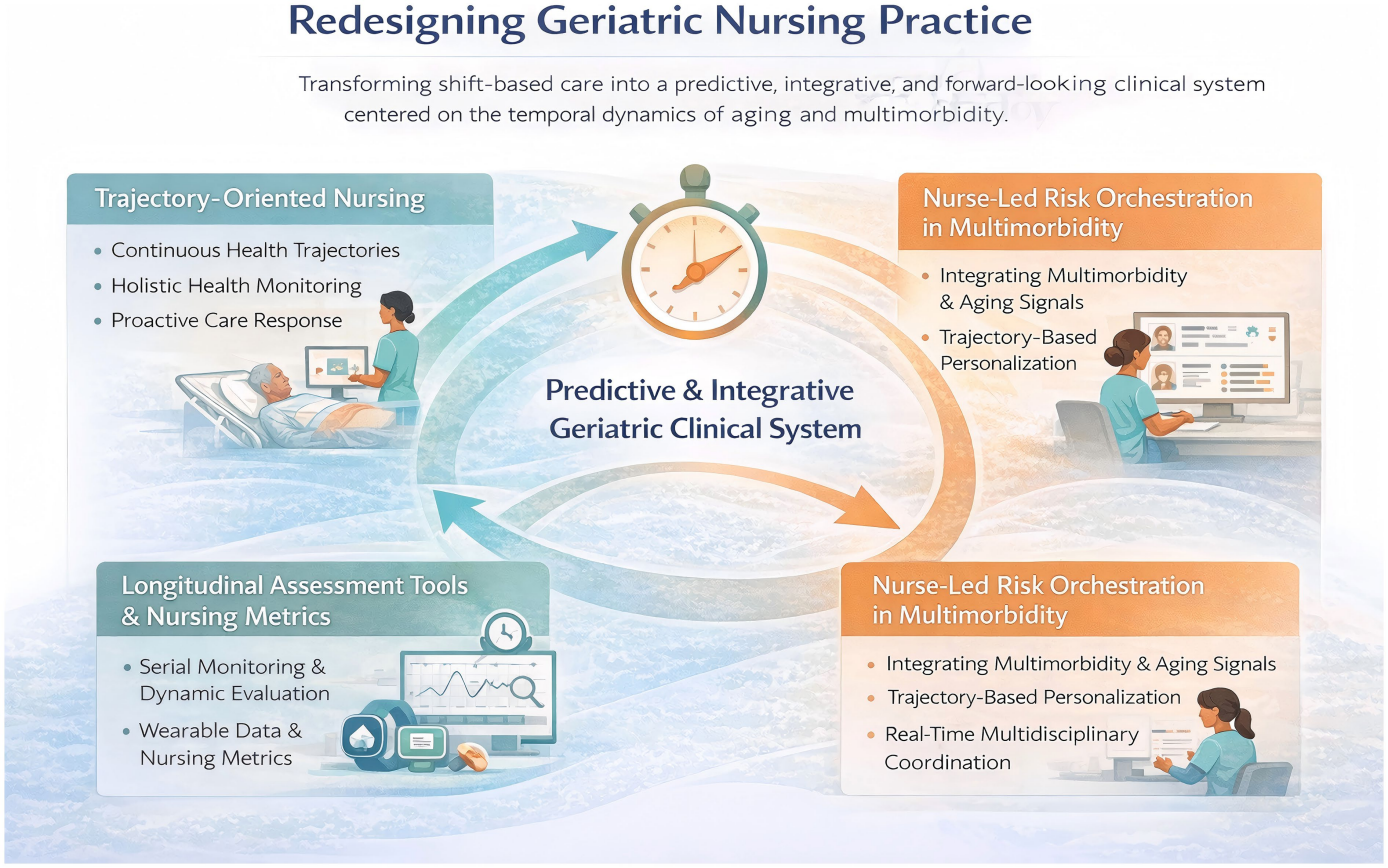
Redesigning geriatric nursing practice through a time-centered clinical framework.

Building on the premise that aging and multimorbidity unfold along continuous, heterogeneous time trajectories, this section translates time-centered nursing theory into concrete clinical practice (Figure 4). Reframing nursing as a temporal discipline necessitates not only conceptual shifts but also structural, methodological, and role-based redesigns within geriatric care. Specifically, effective implementation of time-aware nursing requires (i) reorganizing care delivery from shift-based task execution toward trajectory-oriented surveillance, (ii) adopting longitudinal assessment tools and nursing metrics capable of capturing dynamic patterns of change, and (iii) positioning nurses as active risk orchestrators who integrate multimorbidity, functional decline, and biological aging signals over time. Together, these three dimensions redefine geriatric nursing as a predictive, integrative, and forward-looking clinical system that aligns care decisions with the temporal realities of aging rather than isolated clinical events.

### 5.1 From shift-based care to trajectory-oriented nursing

Contemporary geriatric care remains predominantly structured around episodic, shift-based tasks, where assessments and interventions are framed as discrete events tied to work schedules rather than as continuous processes along the patient’s health trajectory (86). While this model satisfies operational needs, it inadequately captures the multi-dimensional and time-dependent nature of aging and chronic disease progression. Aging is not a sequence of isolated changes; rather, it is a dynamic process with nonlinear trajectories, variable rates of change, and distinct biological inflection points, as underscored by longitudinal omics-based studies demonstrating that molecular and physiological markers evolve in complex patterns across the lifespan (87, 88). For example, comprehensive multi-omics profiling reveals nonlinear changes in molecular phenotypes at critical age transitions, highlighting the inadequacy of single timepoint measurements to detect underlying biological shifts that precede clinical deterioration (87). Similarly, advanced proteomic mapping across multiple timepoints in large adult cohorts identifies longitudinally regulated proteins that reflect aging and disease-associated pathways, underlining the value of temporal trends over snapshots for risk characterization. Longitudinal phenotypic aging metrics further show that accelerated trajectories correlate with faster physical and cognitive decline, multimorbidity accumulation, and shorter survival, outperforming cross-sectional measures of aging phenotype (89). These findings collectively substantiate the core premise that aging and disease progression are best understood and thus best managed through continuous temporal observation rather than isolated measurements.

For nursing practice, such evidence translates into a compelling imperative: shift the focus from episodic checks to trajectory-oriented surveillance. Traditional shift-based care privileges isolated snapshots of patient status (e.g., vital signs or task checklists) that may miss subtle but clinically significant trends, such as gradually declining mobility, incremental cognitive fluctuations, or progressive functional losses that emerge slowly yet portend adverse outcomes (90). In contrast, trajectory-oriented nursing systematically captures repeated, time-anchored observations, providing a holistic understanding of health evolution that aligns with contemporary biological aging research (91, 92). Longitudinal modeling approaches, including machine learning derived biomarkers of aging and resilience dynamics derived from serial physiological measures, demonstrate that temporal patterns contain prognostic information not accessible from cross-sectional data (93). For instance, dynamic organism state indicators derived from longitudinal blood tests reveal age-related loss of physiological resilience long before overt clinical events manifest, reinforcing the need for early detection through repeated measures rather than retrospective diagnoses (94). Moreover, aging clocks constructed from omics and clinical longitudinal data provide refined assessments of biological aging and risk stratification that far exceed traditional age or single biomarker predictions, pointing toward a future where nurses integrate longitudinal biological insights into routine care monitoring (95).

In clinical terms, trajectory-oriented nursing practice entails routinely contextualizing current patient data within their historical trajectory, enabling detection of directional changes (e.g., acceleration or deceleration of decline) that single snapshots cannot reveal. This approach resonates with evidence from studies using longitudinal intrinsic capacity frameworks, which propose quantitative models to map functional aging trajectories as predictors of future health outcomes (96). Nurses equipped with trajectory-oriented frameworks can thus identify deviations from expected aging patterns, anticipate emerging risks, and tailor interventions proactively rather than reactively (97). This paradigm is especially crucial in managing older adults with multimorbidity, where overlapping disease processes and compensatory mechanisms often mask deteriorations until late-stage events. Supporting this perspective, recent research on multimorbidity biomarkers from longitudinal cohorts underscores how shared and specific biological processes accumulate over time, reinforcing the notion that disease burden is temporally structured and context dependent (98). Collectively, this evidence base provides a strong rationale for reframing geriatric nursing away from task-based assessments toward a holistic, temporally informed model that aligns with the underlying dynamics of aging biology and improves predictive capacity for adverse events.

### 5.2 Longitudinal assessment tools and nursing metrics

A fundamental shift in geriatric nursing towards trajectory-oriented care necessitates the development and adoption of longitudinal assessment tools and nursing metrics that capture health dynamics over time rather than static snapshots of function or disease severity. Longitudinal data collection, whether through repeated clinical assessments, biomarker profiling, or continuous digital health monitoring, reveals patterns of change that are more predictive of functional decline, multimorbidity progression, and adverse outcomes than single-timepoint measures (99). For example, longitudinal phenotypic aging metrics have been shown to capture individual rates of biological aging and are associated with faster physical and cognitive deterioration, accumulation of multimorbidity, and shortened survival, outperforming cross-sectional assessments in predictive validity (23). These studies highlight the limitations of one-off snapshots in capturing the complex, non-linear progression of decline in older adults and underscore the necessity for nursing metrics that integrate temporal patterns to detect early deviations from expected trajectories.

Digital health technologies and wearable sensors further expand the capacity for longitudinal assessment with continuous, non-invasive data streams that can be integrated into nursing assessment frameworks (100). Recent advances in wearable based aging clocks, algorithms that estimate biological age from photoplethysmography signals collected passively during daily life, demonstrate how wearables can produce rich longitudinal time series that correlate with disease risk and predict future cardiometabolic events (101). Wearable-derived circadian rhythm metrics, such as the CosinorAge parameter, capture temporal patterns in activity and rest cycles that are strongly associated with healthspan and mortality risk, offering scalable digital biomarkers for longitudinal monitoring (102). Beyond cardiovascular and activity metrics, emerging research shows that passive multimodal wearable data, combining physiological, behavioral, and environmental signals, can predict cognitive and affective outcomes over extended periods, illustrating the feasibility of low-burden continuous tracking of key geriatric domains (103). When these continuous data streams are contextualized within individual health trajectories, nurses can detect subtle early changes such as progressive declines in gait speed, alterations in sleep–wake patterns, or deviations in biological age estimates that may precede clinical manifestations of disease or functional loss.

To translate these longitudinal measurements into actionable nursing metrics, standardized frameworks and analytical models are essential. For instance, integrated aging clocks that combine multiple biomarker domains (epigenetic, proteomic, metabolomic, clinical) within longitudinal pilot cohorts to estimate biological age across multiple time points, enabling personalized health trajectories that inform preventive care decisions (104). In addition, composite instruments such as multidimensional health assessment tools that model organism-specific aging rates across multiple organ systems (e.g., the “Health Octo Tool” integrating Bayesian ordinal regression across 13 systems) illustrate how longitudinal phenotypes can be synthesized into metrics that predict functional decline and mortality with greater precision than traditional frailty indices (105). Importantly, these tools must be interpreted in the context of meaningful clinical change: longitudinal nursing metrics require thresholds and slope parameters that distinguish normal age-related variation from clinically significant deterioration, allowing nurses to stratify risk and escalate care appropriately.

### 5.3 Nurse-led risk orchestration in multimorbidity

Multimorbidity, defined as the concurrent presence of two or more chronic conditions, constitutes a central challenge in geriatric care, profoundly affecting functional capacity, quality of life, healthcare utilization, and mortality (106). In older adult populations, the development and progression of multimorbidity reflect dynamic and heterogeneous trajectories that cannot be fully captured by traditional cross-sectional clinical assessments (107). Beyond trajectories of disease counts, biological and phenotypic aging metrics extracted from longitudinal analyses provide additional depth to risk stratification. Emerging work on biological aging clocks, including proteomic and metabolomic aging profiles, reveals that participant-specific aging acceleration explains a significant proportion of variation in multimorbidity and mortality risk independent of chronological age, thus offering a scalable, biologically grounded set of risk predictors (108). Such integrated biological information affords a deeper comprehension of an individual’s cumulative risk state—knowledge that nurses are uniquely positioned to operationalize by constantly synthesizing clinical, functional, and temporal data. Within this context, nurse-led risk orchestration refers to the proactive integration of multimorbidity trajectories, functional status indices, lifestyle behaviors, psychosocial factors, and aging biomarkers to generate a comprehensive, individualized risk profile that guides personalized care (109). This paradigm transcends traditional dichotomous risk labels (e.g., “multimorbid” vs. “non-multimorbid”) and instead situates patients along continuously evolving risk spectra that are responsive to temporal changes (110). In practice, nurses working longitudinally with older adults can identify early deviations from anticipated health trajectories, such as accelerated declines in physical resilience, emergent functional limitations, or worsening biomarkers of biological aging, and escalate care or modify interventions accordingly (59). In doing so, nurses integrate clinical observation with objective metrics over time, creating a dynamic risk orchestration model that harmonizes care planning with empirically derived trajectory insights.

Operationalizing nurse-led risk orchestration requires multidisciplinary synthesis: functional assessment frameworks (e.g., intrinsic capacity and frailty indices) must interact with biological and behavioral data streams to generate risk scores that evolve with the patient (111). Longitudinal frailty studies show that trajectories of functional decline, when tracked repeatedly, are robust predictors of hospitalization and mortality, suggesting that nurses should prioritize longitudinal frailty assessment as a central metric in risk models (112). Equally, epigenetic and phenotypic frailty risk scores derived from DNA methylation signatures have demonstrated longitudinal predictive validity for frailty progression, reinforcing the relevance of biomarker-linked risk stratification (113). Integrating such biomarkers with comprehensive geriatric assessment, a multidimensional evaluation encompassing clinical, functional, psychological, social, and environmental domains, enables nurses to transcend traditional siloed risk assessment and deliver high-resolution risk orchestration that anticipates points of vulnerability before irreversible decline.

Critically, the nurse’s role as risk orchestrator is not a passive aggregator of data but an active interpreter of time-dependent risk patterns. Nurses integrate information from longitudinal changes in functional capabilities, aging biomarkers, disease clusters, patient-reported outcomes, and psychosocial context to generate composite risk insights that are clinically actionable (114). This interpretive role is essential because it contextualizes quantitative indicators within the lived experience of aging—something that static models or infrequent assessments cannot accomplish. Therefore, nurse-led risk orchestration aligns with personalized medicine by translating high-dimensional data into tailored care strategies, such as early mobilization to counter functional decline, structured cognitive engagement to mitigate neurodegenerative trajectories, or targeted nutritional support to modify metabolic aging (35). In doing so, nurses operate at the intersection of data science, gerontology, and patient-centered care, coordinating interventions that are temporally sensitive, contextually grounded, and prognostically informed.

## 6 Digital and smart technologies as catalysts for continuous, predictive geriatric nursing

**Figure 5:**
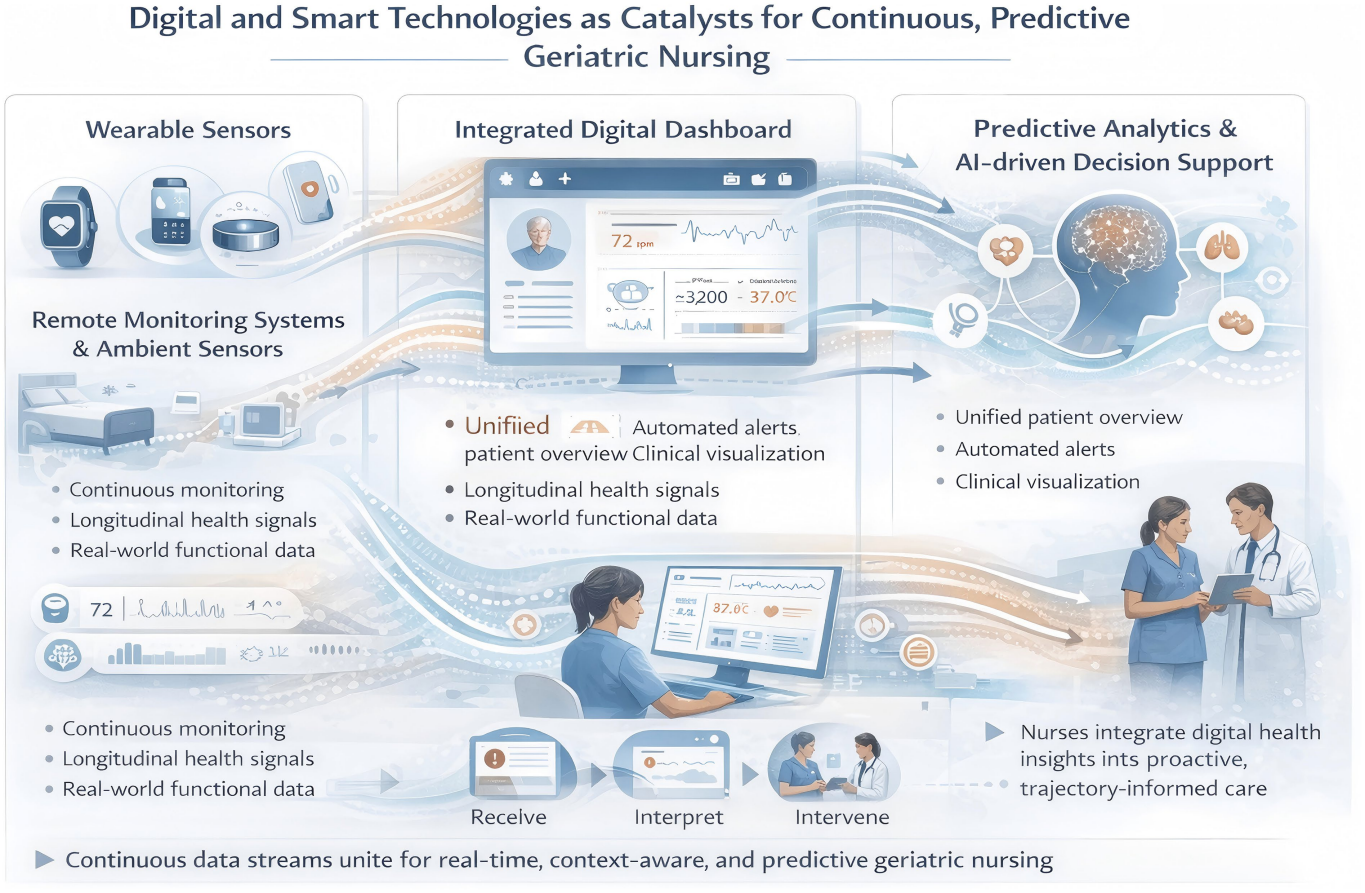
Nurses leverage wearable sensors, integrated dashboards, and AI-driven analytics to transform continuous, longitudinal data into proactive, predictive geriatric care.

The integration of digital and smart technologies is fundamentally reshaping geriatric nursing by enabling continuous, context-aware observation and data-driven anticipatory care. Wearable sensors, remote monitoring systems, and ambient devices capture rich, longitudinal health signals that extend nursing insight beyond episodic assessments, providing early detection of deviations from individual baselines. Coupled with predictive analytics and AI-driven decision support, these technologies transform raw data streams into actionable forecasts of functional decline, multimorbidity progression, and other adverse outcomes. Integrated digital dashboards consolidate heterogeneous data, facilitate interdisciplinary coordination, and enhance nurses’ ability to interpret trends within the patient’s lived experience. Collectively, this digital ecosystem positions nurses not only as monitors but as proactive orchestrators of personalized, trajectory-informed care, bridging continuous surveillance with predictive interventions to optimize health outcomes in aging populations (Figure 5).

### 6.1 Wearable and remote monitoring systems for continuous observation

Emerging wearable and remote monitoring technologies are transforming the landscape of geriatric nursing by enabling continuous, non-invasive observation of physiological, functional, and behavioral health metrics in older adults (115). These technologies shift the traditional model of episodic vital sign checks and periodic assessments toward persistent observation, thereby filling critical gaps in longitudinal health surveillance. A growing body of research highlights that connected sensing devices, such as wearables and body-worn sensors, can collect high-resolution digital measures that serve as proxies for health status or the risk of adverse events, such as mobility decline or falls, outside of clinical settings (116). Such capabilities support a proactive model of care in which deviations from established personal baselines can be detected early and addressed by nurses before they escalate into acute crises (117). The potential utility of this technological paradigm has been underscored in recent reviews of digital health for aging populations, noting that wearables can help older adults track chronic conditions, monitor safety concerns at home, and promote independence through ongoing remote monitoring (100).

At the heart of these innovations are wearable sensors and body area networks that continuously measure vital signs and activity patterns. These include wristbands and smartwatches that capture heart rate, sleep cycles, and step counts; inertial measurement units that quantify gait and balance; and flexible e-textiles embedded with multi-modal sensors to track heart rate, respiration, and temperature directly from clothing (118). The combination of these sensor modalities with body area network architectures enables seamless data collection throughout daily life, creating a dense time series of health signals that reflect real-world status rather than intermittent snapshots (119). For example, recent work has shown that self-supervised learning models applied to wrist-worn accelerometer data can accurately detect gait quality and reflect mobility limitations associated with aging and disease, offering a scalable approach to monitoring important geriatric functional outcomes (120). These technologies, when integrated with remote data platforms, positioned nurses to interpret longitudinal trends rather than isolated values, enabling earlier recognition of health deterioration and more timely interventions (121). Continuous monitoring systems extend beyond physiological signal capture to incorporate comprehensive daily living data through ambient and wearable devices. Open data infrastructures like the RESILIENT platform aggregate information from wearable devices and bedside sensors into unified views of activity, cardiovascular metrics, and sleep patterns, offering healthcare professionals a detailed and longitudinal picture of patient health that can be visualized and acted upon (122). Likewise, remote health monitoring frameworks that integrate sensor networks with decision support systems allow for automated detection of health anomalies and real-time alerts to nurses or caregivers, promoting early intervention and risk mitigation (123). Existing research emphasizes the broad scope of remotely monitored signals, such as heart rate, oxygen saturation, respiratory parameters, activity levels, and sleep patterns, as critical indicators of health trajectory in older individuals (124). Such continuous and multimodal data streams not only enhance the ecological validity of monitoring but also act as the foundation for computational phenotyping and personalized care pathways, ultimately enabling nurses to detect subtle departures from an individual’s baseline and embed trend interpretation into routine care rather than fluently relying on intermittent clinical assessments.

Critically, the success of wearable and remote monitoring systems in geriatric settings depends on user acceptance, device comfort, and sustained adherence. Older adults may face barriers related to technology literacy, physical comfort, and usability, which can impact consistent use and the quality of longitudinal data collected (125). Evidence suggests that older individuals generally show high daily adherence to wearable devices but that sustained use over extended periods may wane, indicating the importance of intuitive design and engagement strategies (126). Addressing these human-factor challenges is essential to ensure that the continuous monitoring paradigm is both feasible and effective in real-world geriatric care environments. Integration with nurses’ workflows and healthcare infrastructures is equally important, as ambient data must be meaningfully contextualized within clinical decision-making processes rather than treated as isolated metrics.

### 6.2 Predictive analytics and AI-enhanced decision support

In the era of digital health transformation, predictive analytics and artificial intelligence (AI) have emerged as foundational technologies for advancing continuous and anticipatory care in older adults (127). Traditional clinical decision making relies on episodic measurements that often lack temporal depth and fail to anticipate future events. In contrast, predictive analytics utilizes longitudinal data streams such as electronic health records, wearable sensor data, and demographic and clinical histories to forecast individuals’ future health trajectories with increasing accuracy (128). Cutting-edge AI systems are capable of simulating health trajectories by learning complex patterns embedded in large datasets, effectively transforming raw health data into individualized prognostic insights (129). A landmark example is Delphi-2 M, an AI model published in Nature that predicts susceptibility to over 1,000 diseases and projects individualized health trajectories decades into the future, using large-scale longitudinal biobank data (130). Such models represent a new paradigm in risk prediction that surpasses single-disease calculators by capturing cross-disease interactions and long-term patterns.

Beyond broad disease prediction, machine learning models have been developed to forecast specific outcomes relevant to aging populations, such as multimorbidity progression and functional decline. Predictive models have also been applied to forecast disability risk, where machine learning-based risk scores integrate demographic, clinical, and lifestyle factors to estimate the probability of future disability, informing early preventive strategies (131). Importantly, longitudinal machine learning approaches can distinguish features of healthy aging from chronic disease risk by modelling entire health trajectories across diverse populations, thereby enabling earlier identification of individuals at risk for adverse outcomes such as loss of independence or mortality (28). In addition to biological data, predictive modeling has been demonstrated to enhance clinical decision support by integrating multimodal data such as demographic information, genetics, and imaging, thereby improving risk stratification and forecasting disease progression, particularly in neurodegenerative diseases (132). These predictive capabilities have profound implications for nursing practice, where real-time risk scores and future state forecasts can guide individualized care plans, prioritize interventions, and support shared decision making.

AI prediction frameworks are also being extended to encompass multidimensional aging constructs such as biological age and organ-specific aging indices, which outperform traditional chronological age and other proxies in predicting all-cause mortality and age-related disease risk (133). A large-scale study leveraging large language models to estimate biological age across more than 10 million individuals illustrated that AI-derived age measures were significantly more predictive of mortality and adverse outcomes than conventional aging metrics, highlighting the transformative potential of AI in risk assessment (134). These AI-powered tools can be integrated into clinical workflows through digital dashboards that synthesize predictive scores with visual risk trajectories, enabling nurses to interpret complex outputs efficiently and make time-sensitive decisions. Predictive risk models have also demonstrated utility in forecasting flexible endpoints associated with hospital activity, such as the likelihood of requiring specialized rehabilitation services or experiencing frailty-related complications, thereby informing discharge planning and care transitions (135). Additionally, predictive analytics grounded in robust longitudinal modeling frameworks, including time-dependent interpretability survival models developed specifically for older adults with multimorbidity, provide dynamic insights into risk evolution over time and help determine optimal timing for intervention (136). Emerging research on synthetic data and digital twins further expands the predictive toolkit available to nurses by generating realistic health trajectories in silico, enabling scenario testing and risk sensitivity analysis without exposing patient data (137). Synthetic data methodologies and digital twin technologies have been proposed as ways to enhance predictive modeling while addressing privacy and interoperability concerns, laying the groundwork for AI systems that can simulate individual future health states under varying care scenarios (138). Although these advanced tools are still under development and require robust validation and ethical governance, their integration with frontline nursing practice offers a future direction where care planning can adapt to individual risk landscapes in near real time.

Predictive analytics and AI-enhanced decision support represent a profound evolution in geriatric nursing, shifting the care model from episodic response to continuous, anticipatory intervention. By coupling longitudinal data streams with robust AI models, nurses can detect early patterns of decline, forecast future risks, and tailor interventions based on individualized predictions rather than one-off clinical snapshots. This integration aligns nursing practice with precision health paradigms and amplifies the nurse’s role as a proactive coordinator of complex geriatric care, ultimately improving outcomes and quality of life for older adults in an increasingly data-rich clinical environment.

### 6.3 Integrated digital dashboards and interdisciplinary coordination

As continuous, multimodal health monitoring becomes increasingly feasible through wearables, ambient sensors, remote data collection, and electronic health records, the central challenge lies in integrating these heterogeneous data streams into coherent, clinically actionable insights that support nurse-led decision making across diverse care contexts (139). Digital dashboards designed for longitudinal health surveillance serve as focal points where disparate physiological, behavioral, and environmental signals converge, enabling dynamic visualization of trends, early detection of deviation patterns, and real-time clinical alerts (140). This integration aligns with broader calls in digital health research to unify continuous data from wearables and remote monitoring into interoperable frameworks that preserve patient privacy, support scalability, and provide clinicians with an intuitive interface for longitudinal patient trajectories. For example, scalable platforms like the Personal Health Dashboard support secure, device-agnostic acquisition, harmonization, and visualization of multi-domain data—from clinical records to wearables—facilitating real-time interpretation at individual and cohort levels (141). These digital interfaces not only aggregate vast data but also translate complex longitudinal patterns into digestible formats that enable nurses to monitor changes over time, recognize early warning signs, and coordinate tailored interventions with interdisciplinary teams.

The integration of dashboards with advanced analytics and interoperability standards is critical for coordinating care among nurses, physicians, specialists, and allied professionals. Digital technologies such as wearables, remote monitoring systems, and cloud-based analytics have the potential to transform geriatric care by enabling continuous health management and supporting independent living among aging populations (100). When integrated dashboards combine longitudinal sensor data with clinical history and predictive models, nurses are empowered to identify subtle deviations, such as changes in sleep patterns, mobility decline, or heart rate variability, that precede clinical deterioration and warrant early, multidisciplinary intervention (72). Dashboards capable of trend visualization with guardrails for anomaly detection thus function as cognitive amplifiers that support nurses’ situational awareness and evidence-based coordination with physicians, rehabilitation specialists, pharmacists, and social workers. Insights from integrated systems, including digital clinical outcome assessments derived from zero-interaction sensor networks, further demonstrate how digital exhaust data can generate comprehensive health summaries that meaningfully augment clinical decisions and risk stratification (117).

Interdisciplinary coordination facilitated by dashboard systems is not limited to real-time monitoring but extends to shared care planning, anticipatory guidance, and longitudinal outcome evaluation. In geriatric care, where multiple chronic conditions intersect with functional decline and psychosocial needs, integrated dashboards help nurses synthesize complex health narratives that span physiological signals, behavioral indicators, medication adherence, and social determinants of health (142, 143). This synthesis enables targeted communication across team members, supporting decisions on referral priorities, home-based interventions, and adjustments to care plans. For instance, integrated dashboards combining physiological data with patient-reported outcomes and environmental sensors can inform collaborative discussions between nurses and physicians about fall risk management or cognitive decline trajectories, with longitudinal visualization clarifying subtle but significant trends over weeks or months (144). The development of interoperable digital health solutions, including AI-powered smart glasses and multimodal interfaces, exemplifies how advanced visualization and interactive analytics can augment clinician engagement and support coordinated action across professional boundaries (145). Furthermore, interoperability efforts that enable unified dashboards to integrate electronic health record data with wearable metrics and behavioral trends help ensure that all stakeholders, including clinicians, caregivers, and patients themselves, operate from a shared understanding of health trajectories, thereby reducing information fragmentation and enhancing care continuity (146).

Beyond clinical benefits, integrated dashboard systems contribute to learning health systems by generating longitudinal datasets that improve predictive models, identify population-level patterns, and inform policy and resource allocation for aging care (147). When dashboards standardize timestamped data streams from continuous monitoring, they create a foundation for machine learning models that can forecast adverse events, such as hospital readmissions, functional decline, or acute exacerbations, well before they occur, thus enhancing nurses’ ability to orchestrate proactive interventions (148). This predictive capacity is reinforced by research showing that wearable sensors and remote monitoring can enable personalized predictions of clinical states, such as laboratory measurements or cognitive/behavioral changes, thereby underscoring the potential for integrated data platforms to extend beyond descriptive dashboards into predictive decision support (149). Importantly, these predictive insights must be contextualized by nurses within the lived experiences and goals of older adults to ensure that care remains person-centered, equitable, and responsive to individual preferences.

Technical interoperability and standards are foundational to achieving this integrated vision. Systems like Personal Health Dashboard, which support device-agnostic data capture and harmonization across wearables, clinical records, and multi-omics data, illustrate how robust digital infrastructure can unify diverse data types into a coherent interface for clinical use (141). Interdisciplinary coordination depends on such infrastructure to facilitate data exchange, ensure security and privacy, and support analytics that are interpretable by clinical end users. In this context, ethical considerations surrounding data governance, model transparency, and user trust become paramount; responsible implementation of dashboard systems requires adherence to privacy protections, patient consent frameworks, and mechanisms for accountable algorithmic decision support (150). The burgeoning field of synthetic data generation and privacy-preserving analytics further highlights how innovative technical solutions can reconcile the need for rich, interoperable datasets with ethical imperatives in geriatric care analytics (151). Integrated digital dashboards act as the nexus where continuous monitoring, advanced analytics, and interdisciplinary collaboration converge to enable nurse-led trajectory-oriented care for older adults. By presenting longitudinal health data in actionable formats, supporting cross-professional communication, and facilitating predictive insights, these technologies extend the nurse’s role as a coordinator of complex care pathways and as a steward of personalized, continuous geriatric care (152). As digital health continues to evolve, the integration of interoperable dashboards with longitudinal datasets and predictive models will be central to realizing the vision of proactive, coordinated, and outcome-driven nursing practice in aging populations.

### 6.4 Implementation barriers and unintended consequences

Despite the promise of time-based and predictive geriatric nursing, real-world implementation remains complex and may introduce unintended consequences if not carefully designed. Beyond ethical principles alone, the implementation of time-based and predictive geriatric nursing also faces substantial practical barriers that may generate unintended harms if insufficiently addressed. First, feasibility at the patient and caregiver level cannot be assumed. Although remote monitoring and wearable systems are often described as low-burden, their real-world use may require sustained device adherence, charging, troubleshooting, repeated data entry, and acceptance of continuous observation, all of which may be difficult for older adults with cognitive impairment, sensory limitations, multimorbidity, or low digital literacy. In some cases, technologies intended to support independence may instead create frustration, dependence on family caregivers, or a sense of being surveilled rather than cared for. Second, these systems may impose additional burdens on nurses and care teams. Continuous data streams, dashboards, and predictive alerts can expand interpretive workload, increase documentation complexity, and disrupt established workflows if outputs are poorly integrated into routine practice. Rather than automatically improving anticipation, excessive or weakly actionable alerts may contribute to alarm fatigue, desensitization, and cognitive overload. False-positive signals may trigger unnecessary assessments, escalation, or anxiety for patients and staff, whereas false-negative predictions may create misplaced reassurance and delay recognition of deterioration. Third, successful implementation depends on organizational capacity, including staffing, training, interoperability, technical support, governance, and leadership commitment. Health systems with limited digital infrastructure or constrained workforce capacity may be unable to translate predictive information into timely action, thereby widening the gap between data generation and meaningful care response. For these reasons, implementation should be approached not as simple technology adoption, but as a socio-technical redesign process requiring workflow co-design, threshold calibration, human oversight, and ongoing evaluation of burden, safety, and equity.

## 7 Beyond continuous care: future directions for geriatric nursing in a time-centric health system

**Figure 6:**
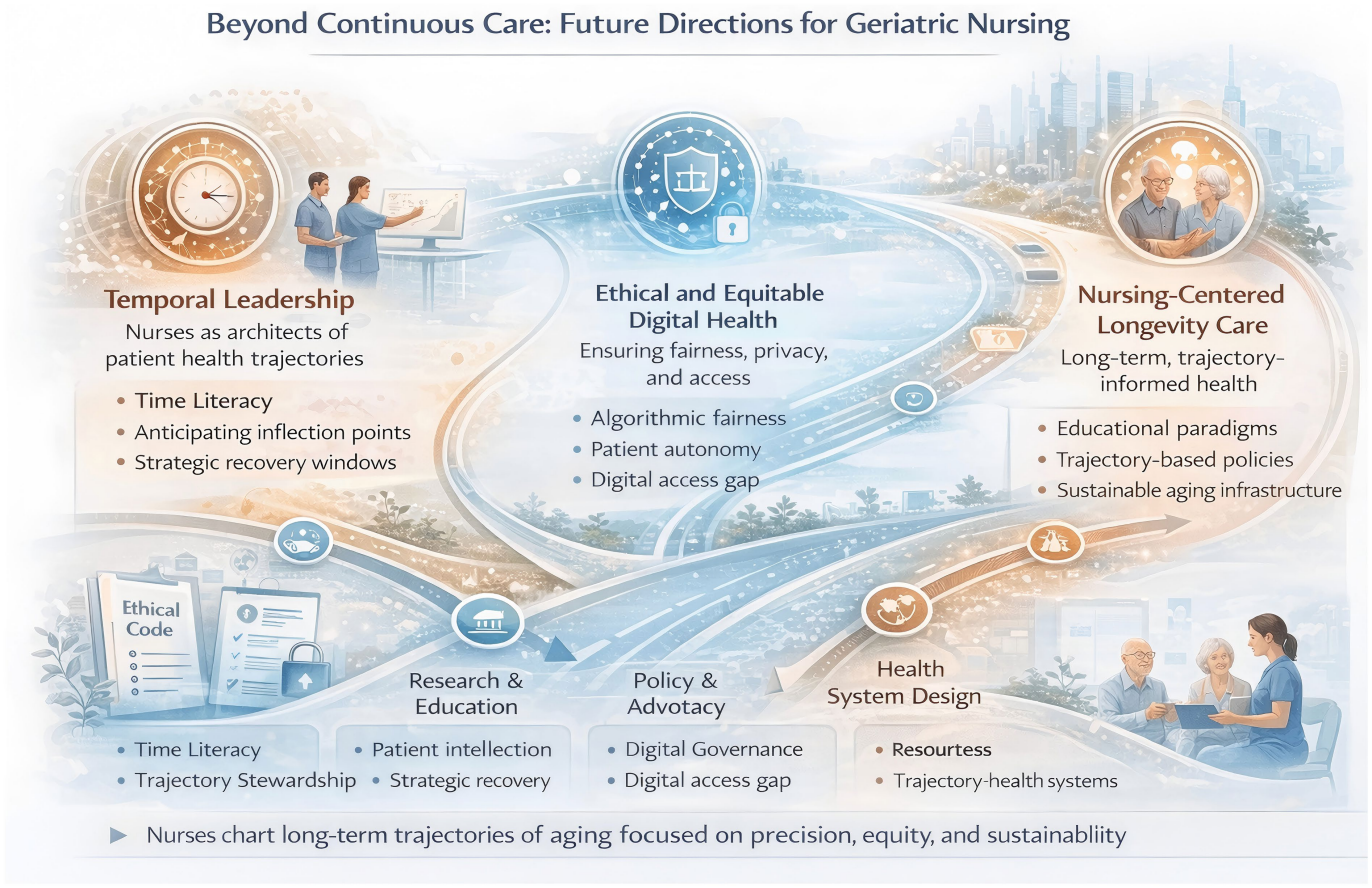
Future directions in geriatric nursing, highlighting nurses as temporal architects, addressing ethical and equity challenges, and advancing a nursing-centered longevity care model through education, policy, and health system design.

Through continuous clinical observation and close patient interaction, nurses may play a key role in identifying early signals of trajectory change and supporting anticipatory care strategies in geriatric practice. Time-centric approaches emphasize the interpretation of longitudinal patterns, identification of critical inflection points, and strategic deployment of interventions during optimal recovery windows. Concurrently, this evolution introduces complex ethical and equity considerations, including algorithmic fairness, patient autonomy, and equitable access to digital health technologies, which must be proactively addressed to ensure just and responsible care. Finally, advancing a nursing-centered longevity care model calls for transformations in education, policy, and health system design, equipping nurses to leverage trajectory-informed decision-making, coordinate interdisciplinary care, and advocate for structures that support sustained healthspan. Together, these developments redefine geriatric nursing as a temporally informed, ethically grounded, and systemically integrated discipline capable of shaping long-term outcomes for aging populations (Figure 6).

### 7.1 From care providers to temporal architects: redefining the epistemic role of nurses

As healthcare systems evolve to manage increasingly complex, chronic, and time dependent conditions, particularly among older adults, traditional roles of nurses as implementers of episodic medical decisions are proving insufficient (153). Historically, nursing practice has prioritized immediate clinical tasks and situation-specific assessments, essentially operating within discrete temporal snapshots of a patient’s health status (154). However, foundational work in health trajectory science demonstrates that understanding change in health and illness over time is central to creating and evaluating interventions that anticipate rather than react to deterioration, placing temporal patterns at the core of clinical insight rather than at the periphery of care processes (155). This shift is especially salient in geriatric contexts, where health outcomes are shaped by overlapping trajectories of multimorbidity, functional capacity, and psychosocial factors that cannot be accurately interpreted at single time points (156). In response, the future of nursing must pivot from viewing care as a series of task completions toward constructing time-cognitive frameworks that structure how health information is interpreted across temporal scales. Such frameworks require nurses to define what constitutes meaningful change, to recognize critical trend patterns, and to intervene at junctures where alterations in trajectory may signal either risk escalation or opportunity for recovery. This epistemic transformation can be described through two emergent concepts: Temporal Literacy in Nursing and Trajectory Stewardship. Temporal Literacy in Nursing refers to the ability of nurses to interpret temporal structures in patient data, understanding not just the presence or absence of symptoms but also the rate of change, inflection points, recovery windows, and periods of vulnerability that characterize health evolution (157). For example, in longitudinal studies of intrinsic capacity or health-related quality of life, researchers identify patterns of stability, acceleration, or deceleration that correlate with later outcomes, suggesting that the temporal shape of change is clinically significant (158). Nurses with temporal literacy would be able to contextualize a subtle but accelerating decline in gait speed or a non-linear pattern of sleep disruption, patterns often invisible in isolated measures, into predictive inferences about functional risk (159). This requires extending nursing knowledge beyond traditional phenomenological assessment toward structured temporal reasoning, such that changes are interpreted not as isolated events but as elements of evolving health narratives. In parallel, Trajectory Stewardship reflects the authority and responsibility of nurses to guide and influence health trajectories, determining which trends warrant intervention and when temporal inflection points present actionable opportunities (160). Rather than solely executing clinical orders, nurses in this role become stewards of patient trajectories, integrating longitudinal observations into care planning, communicating time-based risk to interdisciplinary teams, and advocating for interventions tuned to trajectory context.

Practically, this reconceptualization positions nurses as time interpreters rather than data scribes, a shift that extends traditional professional knowledge into the temporal domain. For instance, longitudinal qualitative methodologies, increasingly employed in health and nursing research to capture participants’ evolving experiences and turning points, illustrate the richness of temporal patterns that static designs overlook (161). Such methodologies validate the premise that nursing outcomes are deeply conditioned by patterns of change in behavioral, functional, and psychosocial domains over time. This validation underscores the need for temporal literacy as a core competency: nurses must discern not just that a change has occurred, but how the tempo, direction, and context of that change relate to future risk and resilience (162). Temporal literacy thus becomes a foundational skill that enables nurses to translate longitudinal observations into prognostic understanding, bridging the gap between raw temporal data and meaningful clinical action. Furthermore, trajectory stewardship elevates nursing influence in decision activation—the moment when temporal interpretation informs intervention choice. A nurse with expertise in trajectory stewardship would recognize, for example, a subtle slowing of cognitive reaction times across successive assessments as an early warning of neurodegenerative acceleration, prompting multidisciplinary engagement before clinical thresholds are crossed (163). Likewise, understanding recovery windows, periods when physiological systems are responsive to intervention, allows nurses to time rehabilitative or preventive measures for maximal effect, a capability that static assessment frameworks cannot reliably support (164). Importantly, this stewardship role does not replace medical decision making but rather augments it by embedding time into the very logic of care decisions, ensuring that interventions are temporally aligned with individual health dynamics rather than tethered to episodic clinical markers.

To improve operational clarity, these concepts should be understood as preliminary practice-oriented constructs that can be translated into observable nursing competencies and implementation processes. Temporal Literacy in Nursing refers to the nurse’s ability to recognize and interpret longitudinal patterns of change across repeated assessments, including direction, slope, variability, acceleration or deceleration, inflection points, and recovery windows. This competency may be examined in future work through longitudinal case vignettes, simulation-based assessment, or standardized scenario interpretation. Trajectory Stewardship refers to the nurse’s role in incorporating temporal change into care planning, clinical escalation, and adjustment of intervention timing; its observable manifestations may include trend-based documentation, earlier recognition of deterioration, and trajectory-informed interdisciplinary communication. Nurse-led risk orchestration refers to the dynamic integration of multimorbidity, frailty or intrinsic capacity trajectories, patient-reported outcomes, psychosocial context, and relevant biomarker or digital monitoring data into an individualized and updatable risk profile. These constructs are not intended to replace established geriatric nursing competencies such as comprehensive assessment or care coordination, but to extend them by emphasizing time-sensitive interpretation, longitudinal synthesis, and anticipatory intervention. Future validation may include expert consensus methods, competency framework development, inter-rater reliability testing using standardized longitudinal cases, and prospective studies examining associations with earlier intervention and geriatric outcomes.

### 7.2 Ethical and equity challenges in time-based geriatric nursing

As geriatric nursing evolves toward predictive, time-centric care models, leveraging longitudinal data streams and advanced analytics, ethical considerations become paramount. Integrating continuous monitoring, risk prediction algorithms, and real-time decision support into care workflows can improve early detection of decline and personalize interventions (165). However, these predictive technologies also introduce multidimensional ethical and equity challenges that must be addressed proactively to prevent inadvertent harms. One primary concern is algorithmic fairness and bias: predictive models trained on historical clinical data may perform unevenly across demographic groups, leading to misclassification and inequitable allocation of care resources (166, 167). For example, machine learning models predicting Alzheimer’s disease progression have demonstrated significant disparities in sensitivity between Hispanic, Black, and White participants, suggesting that model outputs could disadvantage underrepresented groups if deployed without careful fairness assessment (168). In the broader context of healthcare AI, systematic algorithmic bias has been identified as a risk that can undermine equitable care delivery, driven by imbalances in training data and by historical inequities manifested within clinical datasets (169). This challenge is central to time-based nursing applications because biased trajectory prediction may lead to differential risk stratification, with some patients labeled “high risk” prematurely and others overlooked, thereby exacerbating health disparities rather than ameliorating them. Closely related to algorithmic fairness is the ethical issue of transparency, explainability, and accountability in predictive systems. Predictive models used in longitudinal care often function as “black boxes,” generating risk scores without clear rationale for nurses or patients (170). In healthcare more broadly, the opacity of AI systems has been critiqued for undermining trust and complicating informed consent, especially when patients cannot understand how their data are used or how predictions are generated (171). Without sufficient explainability, nurses may be forced to act on algorithmic guidance without fully understanding its basis, chipping away at professional autonomy and potentially conflicting with ethical principles like do no harm (172, 173). Furthermore, integrated predictive systems must navigate privacy and data responsibility concerns, particularly in older populations where sensitive longitudinal health data (e.g., cognitive function, mobility patterns, psychosocial assessments) may be continuously collected and stored (174). Ethical commentators highlight privacy risks inherent to digital health technologies for older adults, noting that without robust governance frameworks, pervasive data capture can infringe on autonomy and confidentiality while contributing to digital surveillance that older adults may neither fully understand nor consent to Finco et al. (175).

Beyond algorithmic fairness and transparency, equity of access and the digital divide pose considerable ethical issues for time-based geriatric nursing. Technologies underpinning continuous care, including wearables, ambient sensors, and telemonitoring, are not equally accessible across socioeconomic and geographical contexts and may therefore privilege individuals with greater resources and digital literacy while leaving disadvantaged older adults behind (176, 177). Rapid reviews of telerehabilitation and other digital interventions underscore how disparities in internet access, device ownership, and technical support can translate into inequitable service delivery, with economically marginalized and rural older adults at risk of receiving suboptimal care (178). In addition, ethical analyses of smart health technologies reveal recurring concerns about autonomy and responsibility; for example, systematic reviews of smart home health systems for older adult care identify privacy, autonomy, trust, and ageism as core ethical categories that require careful consideration in deployment (179). Thus, geriatric nursing must go beyond technological adoption to advocate for inclusive infrastructure, equity-focused implementation strategies, and culturally sensitive design to ensure that predictive longitudinal care enhances rather than detracts from equity in health outcomes. Another layer of ethical complexity arises from professional role and responsibility attribution in predictive spaces. As nursing decision support incorporates algorithm-generated risk trajectories, clear delineation of responsibility between clinicians and technology becomes crucial (180, 181). The “ethical governance” literature in nursing highlights risks related to professional autonomy when predictive tools influence clinical decisions without transparent accountability pathways (182). Nurses must be empowered to question, interpret, and override predictive suggestions where appropriate, avoiding over-reliance on automated outputs that could undermine human judgment and patient-centered care—a principle echoed across ethical discussions of digital health (183). Furthermore, predictive models may inadvertently shift ethical burdens onto nurses, requiring them to reconcile conflicting signals from machine outputs and individual patient contexts, especially when social and contextual factors such as cultural preferences, socioeconomic constraints, or caregiver availability modulate the interpretation of risk scores (184).

Finally, predictive, continuous care paradigms raise broader ethical questions about patient autonomy, consent, and the right to one’s temporal health narrative. Continuous data collection and longitudinal risk profiling can infringe on perceived autonomy if patients feel surveilled rather than supported, particularly when algorithmic systems intervene without explicit understanding by the patient (185). Ethical analyses of digital health in aging underscore that respect for autonomy and informed consent must be central to design and deployment, requiring transparent communication and shared decision-making frameworks that honor older adults’ values (186, 187). Moreover, digital health ethics in older adult care highlight that governance structures must protect individuals from being reduced to algorithmic risk profiles, preserving human dignity and agency within increasingly data-rich care environments (175). This requires nursing leadership in shaping policy, oversight, and education, ensuring that technological innovation supports equity, respect, and ethical integrity rather than merely accelerating data collection and prediction capabilities without adequate moral guardrails.

### 7.3 Toward a nursing-centered longevity care model: implications for education, policy, and health systems

The evolution of geriatric nursing toward continuous, predictive, and trajectory-informed care invites a broader rethinking of how health systems conceptualize and deliver long-term care for aging populations. In a future shaped by longevity science and digital health integration, nurses are positioned not merely as executors of episodic clinical decisions, but as central coordinators of a nursing-centered longevity care infrastructure—a model that elevates nursing roles across education, policy, and health systems design (188). Aging is increasingly recognized as a modifiable biological process rather than an inevitable decline, propelled by discoveries in geroscience indicating that interventions targeting molecular and physiological hallmarks of aging (e.g., senescence, chronic inflammation, metabolic dysfunction) can influence healthspan and reduce risk for multiple age-related diseases simultaneously (189, 190). For example, interventions such as senolytic therapies that target cellular senescence have shown the potential to improve physical function and delay multimorbidity in preclinical models, illustrating how aging biology insights may reshape preventive care strategies in older adults (191). Within this scientific landscape, nursing must adapt by developing curricula and competencies that encompass not only clinical tasks but also longevity-oriented risk trajectories, health system navigation, and interdisciplinary leadership (192). Educational reforms that integrate geroscience literacy, longitudinal data interpretation skills, and trajectory stewardship will be essential to prepare nurses for roles that influence policy, guide resource allocation, and advocate for trajectory-driven care models rather than episodic service delivery (193). Indeed, calls for incorporating systems thinking into health professions education emphasize that future clinicians, including nurses, must be equipped to operate across scales of care, from individual biological trajectories to population health dynamics.

Building on this educational foundation, health systems must evolve to support nurse-led longevity care pathways that align with trajectory-based reimbursement, integrated service delivery, and cross-sector coordination (194). Traditional payment models that reimburse episodic encounters and discrete procedures are poorly suited to continuous longitudinal care; instead, value-based and capitation frameworks that incentivize long-term functional maintenance and risk mitigation are more consistent with longevity-oriented nursing practice (195). For instance, accountable care models that reward successful management of chronic disease burden, measured not only by disease counts but also by trajectory stability and improvement, could empower nurses to implement early interventions that reduce costly hospitalizations and improve quality of life (196). Evidence from health economics research indicates that proactive, longitudinal care coordination reduces emergency utilization and total cost of care in aging populations, supporting transition to models that compensate sustained preventive efforts (5). Moreover, pilot programs integrating community-based nursing care with digital monitoring and predictive analytics have reported improvements in functional outcomes and reductions in readmissions, underscoring the feasibility of nurse-centered longevity models when supported by appropriate infrastructure (197). Health systems that embed nurses as central decision-makers in care navigation platforms, population health teams, and longevity clinics can harness the full potential of continuous data while preserving human judgment and ethical oversight (198). Cross-disciplinary collaboration encompassing medicine, rehabilitation, social services, and geroscience research will be critical for operationalizing longevity care pathways that respond to individual risk trajectories and socio-environmental contexts.

Policy frameworks must parallel educational and system redesign to ensure that nursing contributions to longevity care are structurally supported and equitably distributed. Policymakers can facilitate this transformation by establishing clinical standards and quality metrics that reflect trajectory changes (e.g., slowing functional decline, stabilizing frailty scores) rather than isolated clinical events (199). International policy efforts to define intrinsic capacity and functional aging as core outcomes illustrate the global movement toward metrics that resonate with longevity goals, and nursing input is integral to refining and implementing these measures across care settings (97). Social determinants of health, such as socioeconomic status, education, and access to technology, must be integrated into policy levers to ensure that longevity care models do not exacerbate existing health disparities. Research in social epidemiology highlights how disadvantage accumulates over the life course to shape health trajectories in older age, suggesting that policies aimed at mitigating inequity upstream (e.g., education, housing, nutrition) can influence later life trajectories and reduce the burden on care systems (200, 201). Nurses, given their patient-proximate position and contextual insight, are well-placed to advocate for policies that integrate trajectory-sensitive social risk screening into care planning and resource allocation. In addition, interoperability standards that ensure seamless data flow across electronic health records, wearable platforms, genomics, and social care systems will be necessary to operationalize longevity care models at scale (202). Studies on health information exchange demonstrate that robust interoperability improves care continuity, especially for populations requiring complex, longitudinal coordination such as older adults with multimorbidity. Importantly, nursing leadership in longevity care will also involve ethical stewardship of predictive technologies and longitudinal data governance. As care models rely on long-term monitoring and predictive analytics, policies must protect patient autonomy, privacy, and consent over prolonged engagement with digital systems, ensuring that older adults retain control over their temporal health narratives (203, 204). Ethical frameworks proposed for continuous digital health interventions emphasize transparency, fairness, and accountability—principles that nurses can champion in multidisciplinary policy dialogues (169). Additionally, emerging evidence on community-engaged design of health technologies underscores that involving older adults in co-creation improves usability and trust, supporting equitable uptake of longevity care innovations (205). In this way, longevity care extends beyond clinical outcomes to embrace participatory care design, where nurses serve as mediators between technological promise, patient values, and societal expectations.

## 8 Conclusion

The growing burden of ageing and multimorbidity exposes a fundamental misalignment between the temporal nature of geriatric disease and the episodic structure of contemporary care systems. Geriatric nursing may represent an important clinical interface for integrating longitudinal patient observation with emerging predictive health approaches, potentially supporting earlier identification of health deterioration in ageing populations. As this review has shown, functional decline in older adults unfolds as a prolonged, dynamic, and often subclinical process, in which clinically meaningful changes emerge long before acute events or diagnostic thresholds are reached. Within this context, nursing continuity represents a critical yet undervalued source of clinical intelligence. Through sustained engagement across care settings and time, nurses are uniquely positioned to detect subtle deviations from individual baselines, integrate multidimensional signals, and translate longitudinal trends into anticipatory interventions. This capability reframes geriatric nursing from a task-oriented, reactive function into a form of time-based clinical reasoning, where value lies in trajectory interpretation rather than isolated actions. Advances in digital health and predictive analytics further amplify this role by enabling continuous monitoring and temporal pattern recognition; however, their impact depends on nursing-led interpretation, ethical governance, and contextual judgment. Without such integration, data abundance risks reinforcing fragmentation rather than improving outcomes. Embedding time continuity at the core of geriatric nursing therefore has profound implications for care delivery, workforce development, and health policy. Recognizing nurses as stewards of health trajectories offers a scalable pathway toward more preventive, equitable, and sustainable ageing care, shifting the focus of health systems from responding to late-stage events to shaping the course of ageing itself over time. Future empirical studies are required to determine how trajectory-oriented nursing concepts can be operationalized and validated in real-world clinical settings.
